# Layered classification in Parkinson’s disease: a content analysis of clinical, prodromal, and biological criteria evolution

**DOI:** 10.3389/fnagi.2026.1935021

**Published:** 2026-08-20

**Authors:** Aishanjiang Yusufujiang, Shan Zeng, Hongyan Li

**Affiliations:** 1 Department of Neurology, People’s Hospital of Xinjiang Uygur Autonomous Region, Urumqi, Xinjiang, China; 2 Xinjiang Clinical Research Center for Stroke and Rare Neurological Disease, Urumqi, Xinjiang, China

**Keywords:** alpha-synuclein, biomarkers, content analysis, diagnostic criteria, Parkinson’s disease, prodromal Parkinson’s disease

## Abstract

**Background:**

Parkinson’s disease (PD) is no longer described through a single diagnostic vocabulary. Clinical criteria, prodromal probability models, and biological research classifications now operate in parallel after the 2024 NSD-ISS and SynNeurGe proposals.

**Objective:**

To clarify how major PD criteria and research frameworks have justified diagnostic change and redistributed the roles of clinical signs, biomarkers, genetics, and prodromal markers.

**Methods:**

We conducted a targeted document-based qualitative content analysis of eight purposively selected landmark PD criteria or criteria-adjacent framework documents, with the 1988 UK Brain Bank/Lewy body source retained as a historical lineage anchor. Document-declared aims and perceived diagnostic problems were coded with a prespecified framework, and twelve diagnostic elements were assigned interpretative ordinal scores according to their document-specific textual roles.

**Results:**

Within the selected corpus, stated priorities shifted from clinicopathological specificity, standardization, and differential diagnosis toward early identification, biomarker integration, staging/subtyping, and trial readiness. Diagnostic-element roles moved from motor signs, exclusions, and dopaminergic response toward RBD/olfaction in prodromal probability frameworks and toward α-synuclein SAA, dopaminergic imaging, and genetics in 2024 biological research frameworks. The analyzed documents indicated materially different 2024 architectures: NSD-ISS emphasized biological definition and integrated staging, whereas SynNeurGe used a multidimensional S/N/G structure.

**Conclusion:**

Our analysis suggests that contemporary PD classification can be understood as a layered architecture rather than a simple replacement sequence. Clinical criteria remain necessary for care, prodromal criteria support early-risk research, and biological frameworks require longitudinal, pathological, technical, ethical, and global-access validation before clinical translation.

## Introduction

Traditionally, the identification of Parkinson’s disease (PD) has rested on bedside clinical assessment, including motor parkinsonism, exclusion of competing diagnoses, dopaminergic responsiveness, and, ultimately, clinicopathological confirmation (Gelb et al., 1999; Gibb and Lees, 1988; Hughes et al., 1992; Jankovic, 2008; Postuma et al., 2015). That approach still governs patient-facing diagnosis, but an expanded research vocabulary has emerged alongside it, encompassing prodromal probability, α-synuclein seed amplification assays (SAA), dopaminergic imaging, genetic stratification, biological staging, and at-risk states (Agin-Liebes et al., 2025; Cardoso et al., 2023; Coughlin et al., 2025; Fernandes Gomes et al., 2023; Höglinger et al., 2024; Kalia et al., 2024; Orrú et al., 2025; Siderowf et al., 2023; Simuni et al., 2024). The 2024 NSD-ISS and SynNeurGe proposals made this tension explicit. Rather than merely updating a clinical checklist, they describe research populations that may precede, overlap with, or partly diverge from the classical clinical syndrome (Höglinger et al., 2024; Simuni et al., 2024).

This transition is often compared with biomarker-driven redefinition in Alzheimer’s disease, where research criteria increasingly distinguish biological disease from the clinical syndrome (Dubois et al., 2007; Jack et al., 2018). That comparison is informative but incomplete. In PD, α-synuclein biology, dopaminergic neurodegeneration, genetic risk, non-motor prodromes, and clinical parkinsonism do not form a single ordered sequence. Lewy body pathology remains historically important, yet some genetic and clinical subgroups complicate a purely Lewy-centered model. A PD framework that begins with biological evidence therefore changes more than test selection. It changes what counts as the research object, who is enrolled into trials, and how uncertainty is communicated to people without clinical PD.

Recent PD reviews and viewpoints cover important parts of this debate. Marsili and colleagues traced diagnostic concepts from James Parkinson to prodromal disease, whereas Reyes and colleagues compared the architecture of the two emerging biological frameworks (Marsili et al., 2018; Reyes et al., 2025). These narrative and comparative accounts, together with MDS and post-2024 commentaries, emphasize that biological frameworks are research tools rather than routine clinical criteria (Cimmino et al., 2025; Höglinger and Lang, 2024; Kalia et al., 2024; McNamara et al., 2026; Reyes et al., 2025). SAA reviews and cohort studies explain why α-synuclein detection has become central and identify constraints in assay standardization, tissue source, access, and false positives outside classical synucleinopathies (Agin-Liebes et al., 2025; Bentivenga et al., 2024; Coughlin et al., 2025; Fernandes Gomes et al., 2023; Grossauer et al., 2023; Orrú et al., 2025; Siderowf et al., 2023; Simuni et al., 2024; Zhao et al., 2025). They do not, however, systematically code how the criteria and framework documents themselves justified each change or redistributed operational authority among symptoms, exclusions, imaging, α-synuclein assays, genetics, cognition, olfaction, RBD, and dopaminergic response.

**FIGURE 1:**
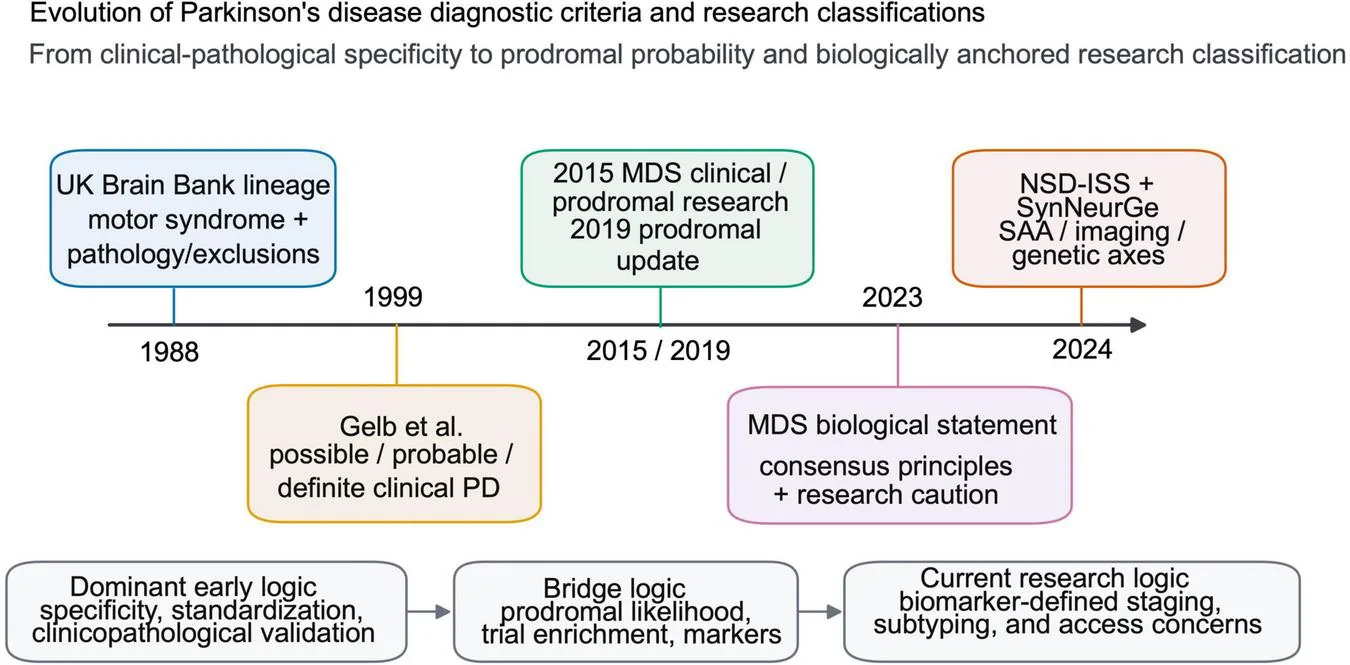
Evolution of Parkinson’s disease diagnostic criteria and research classifications. Schematic overview of the transition from clinical-pathological specificity and clinical standardization toward prodromal probability and biologically anchored research classification. The 2024 NSD-ISS and SynNeurGe frameworks are shown as research classification frameworks rather than routine clinical replacements. SAA, seed amplification assay.

We therefore treated major PD criteria and criteria-adjacent frameworks as documents that do two things at once: classify disease and explain why classification had to change. The analysis had three linked tasks. We identified the documents connecting the UK Brain Bank/Lewy body lineage to current biological research frameworks. We then coded the document-declared aims and diagnostic problems. Finally, we compared how selected diagnostic elements were positioned across clinical, prodromal, and biological frameworks. We used those findings to examine disease boundaries, clinicopathological validation, natural-history estimates, trial enrichment, and disclosure ethics. In this sense, criteria evolution in PD is not only a technical change in diagnostic tools; it is a nosological change in the boundary between disease, risk, prodrome, and research-defined biological state (Figure 1).

## Materials and methods

### Study design and corpus construction

This was a targeted document-based qualitative content analysis, not a systematic review of all PD diagnostic studies. PubMed verification searches were used to check coverage of the literature space and to identify relevant commentaries or validation studies; they were not used to generate a PRISMA-screened evidence base. The findings and code frequencies therefore describe the purposively selected landmark corpus rather than the entire PD diagnostic literature. Three PubMed search blocks were run: a broad criteria/diagnosis block (1983–2025), a commentary/critique block (1983–2025), and a recent biological-framework/biomarker block (2020–2026). Supplementary Methods 1 provides the search strings, run date, hit counts, and sample PMIDs.

The analytic corpus was a purposively selected set of landmark PD criteria and criteria-adjacent framework documents: Gibb and Lees/UK Brain Bank lineage (1988), Gelb et al. (1999), MDS clinical diagnostic criteria (2015), MDS prodromal criteria (2015), the MDS prodromal update (2019), the MDS biological-definition statement, NSD-ISS, and SynNeurGe. We included documents that shaped the definition, diagnosis, staging, or research classification of PD or PD-related biological disease states. Documents focused only on atypical parkinsonian syndromes, rating scales, conference abstracts, unavailable full texts, or biomarker validation without a criteria/framework role were excluded from the primary coding corpus. The 1988 source underwent the same passage and role coding and was included in the reported code-frequency distributions, but its contribution was interpreted cautiously because it served as a lineage/pathology anchor rather than a formal criteria-development consensus paper. Commentary and validation studies informed contextual interpretation in the Introduction, Results, and Discussion but were not coded as primary criteria documents.

### Qualitative content analysis

We used directed qualitative content analysis with a prespecified coding framework, informed by Hsieh and Shannon’s content-analysis typology (Hsieh and Shannon, 2005). Coding categories were applied to textual evidence extracted from the documents, rather than inferred from secondary summaries. PDFs were converted to plain text. Candidate passages were first located with terms that often signal aims or problems, including aim, purpose, objective, need, designed to, limitation, challenge, concern, unclear, insufficient, risk, misdiagnosis, and false positive/false negative. These terms were retrieval aids, not coding rules. A passage could be included without a trigger term if it explicitly stated the purpose, intended use, limitation, or diagnostic problem of the criteria/framework. Background scientific statements were excluded unless the document linked them to diagnostic purpose. Supplementary Table 1 reports the passage-level evidence, including both trigger-term and non-trigger-term inclusions, to permit document-by-document verification.

### Aims/problems coding

Eligible passages were coded with prespecified A1-A10 and P1-P10 schemes. Multiple codes per passage were allowed. Aim codes covered diagnostic specificity, sensitivity, early/prodromal diagnosis, biomarker integration, clinical applicability or operationalization, disease-modifying therapy development, cross-population applicability, staging/subtyping, reduction of misdiagnosis, and unification/simplification. Problem codes covered insufficient specificity, insufficient sensitivity, subjectivity, delayed diagnosis, lack of biomarker integration, complexity, cross-population validity limits, clinicopathological mismatch, early-trial limitations, and unclear prodromal/asymptomatic classification. Supplementary Table 1 provides the passage-level coding.

### Diagnostic-element role scoring

We used a purposively selected comparative panel of twelve diagnostic elements rather than an exhaustive taxonomy of PD manifestations or biomarkers. The panel was chosen to span three domains that could be followed across successive documents: established clinical and exclusionary criteria, prodromal or supportive markers, and emerging imaging, molecular, tissue, and genetic anchors. Selection also required a discernible criterion- or framework-level role in at least part of the corpus and sufficient conceptual continuity for cross-document comparison. Autonomic manifestations were included within non-motor symptoms, and dopaminergic PET and SPECT were grouped under dopaminergic imaging. Emerging domains such as digital biomarkers were not scored separately because they had not acquired a sufficiently stable operational role across the included documents.

The twelve elements were motor symptoms, non-motor symptoms, exclusions/red flags, DAT-SPECT or dopaminergic imaging, MRI, CSF α-synuclein SAA, skin α-synuclein assay/biopsy, genetics, neuropsychological/cognitive assessment, olfaction, RBD, and dopaminergic response. Scores were 0 = absent, 1 = supportive/contextual, 2 = important, and 3 = core/defining. These were document-specific interpretative ordinal judgments of textual function, not objective quantitative measurements; they do not estimate diagnostic accuracy, evidence quality, clinical importance, assay performance, or routine-use endorsement, and the intervals between categories were not assumed to be quantitatively equivalent. We assigned 3 when an element was required to define, classify, stage, or positively identify the entity in that document or framework. For biological research frameworks, “core/defining” includes core research-classification or staging anchors and should not be read as a routine clinical diagnostic requirement. A score of 2 indicated a major but not independently defining role, including likelihood-ratio components, framework anchors, or major red-flag/exclusion categories. A score of 1 indicated contextual, supportive, indirect, or example-level relevance. Supplementary Methods 2 documents the detailed scoring rules and non-intuitive decisions, including the 2015 MDS role of normal presynaptic dopaminergic imaging as an absolute exclusion rather than a positive PD-specific biomarker.

### Independent blinded expert review and adjudication

Primary extraction and coding were performed by Aishanjiang Yusufujiang (A.Y.), a movement-disorders specialist. Shan Zeng (S.Z.), a second movement-disorders specialist blinded to the primary coding, independently reviewed the extracted passages, aim/problem code assignments, and diagnostic-element role scores. Disagreements and boundary judgments were adjudicated by Hongyan Li (H.L.), a third movement-disorders specialist, through source-based review of the relevant passages and coding rationales. Conservative adjudication rules were applied: multi-label aim/problem codes were retained only when explicitly supported by the quote or its immediate document context, and ordinal role scores were assigned according to criterion-level function rather than general biological importance. The Results tables report the adjudicated coding. Further details of the independent review and adjudication are provided in Supplementary Methods 3.

## Results

### Corpus overview and coding distribution

The corpus contained three clinical-lineage/clinical criteria documents, two prodromal research criteria documents, one biological-definition statement, and two 2024 research classification frameworks. Code assignments were non-mutually exclusive: one passage could receive more than one aim or problem code. Within this corpus, staging/subtyping (A8) and biomarker integration (A4) were the two most frequent aim categories, followed by unification/simplification (A10), early/prodromal diagnosis (A3), and disease-modifying therapy development (A6). Problem coding was led by unclear prodromal/asymptomatic classification (P10), insufficient specificity/misdiagnosis (P1), insufficient sensitivity/missed diagnosis (P2), and inability to support early clinical trials (P9). The 1988 historical anchor was included in these counts but interpreted cautiously. Full by-document code-frequency distributions are reported in Supplementary Table 3 and visualized in Supplementary Figure S1; the passage-level assignments appear in Supplementary Table 1. Table 1 provides a condensed synthesis of document types, primary stated motives, key content shifts, and cautions.

**TABLE 1:** Condensed criteria-evolution synthesis.

Document	Document type	Primary stated motive	Key content shift	Caution
Gibb and Lees (1988) UK Brain Bank lineage	Lineage/pathology anchor	Clinicopathological specificity	Lewy body/pathology anchoring and motor/exclusion checklist lineage	Contextual rather than a standalone criteria-development consensus paper
Gelb et al., 1999	Clinical diagnostic criteria	Explicit clinical standardization	Possible/probable/definite categories; motor features and alternative-diagnosis features	Clinical classification, not biological definition
MDS clinical criteria (2015)	Clinical diagnostic criteria	Calibrated clinical probability	Prerequisite parkinsonism plus supportive criteria, absolute exclusions, and red flags	Biomarkers remain supportive/exclusionary, not defining
MDS prodromal research criteria (2015)	Prodromal research criteria	Early/prodromal risk identification	Likelihood-ratio model using RBD, olfaction, subtle motor signs, autonomic and other markers	Research likelihood/enrichment framework, not routine diagnosis
MDS prodromal criteria update (2019)	Prodromal research criteria update	Update prodromal prediction evidence	Updated marker likelihood ratios and risk estimates	Still research-use and probabilistic
MDS biological definition statement (2023)	Biological-definition statement	Consensus principles for biological definition and staging	Foundational principles and explicit research-only caution	Not a final operational diagnostic replacement
NSD-ISS (2024)	Research staging/classification framework	Biological definition and integrated research staging	Neuronal α-synuclein disease staging with SAA and dopaminergic dysfunction anchors	Research framework; does not replace clinical PD diagnosis
SynNeurGe (2024)	Research diagnostic/classification framework	Multidimensional biological classification	S/N/G axes: synuclein pathology, neurodegeneration, genetics	Research framework; do not frame as formal conflict with NSD-ISS

**FIGURE 2:**
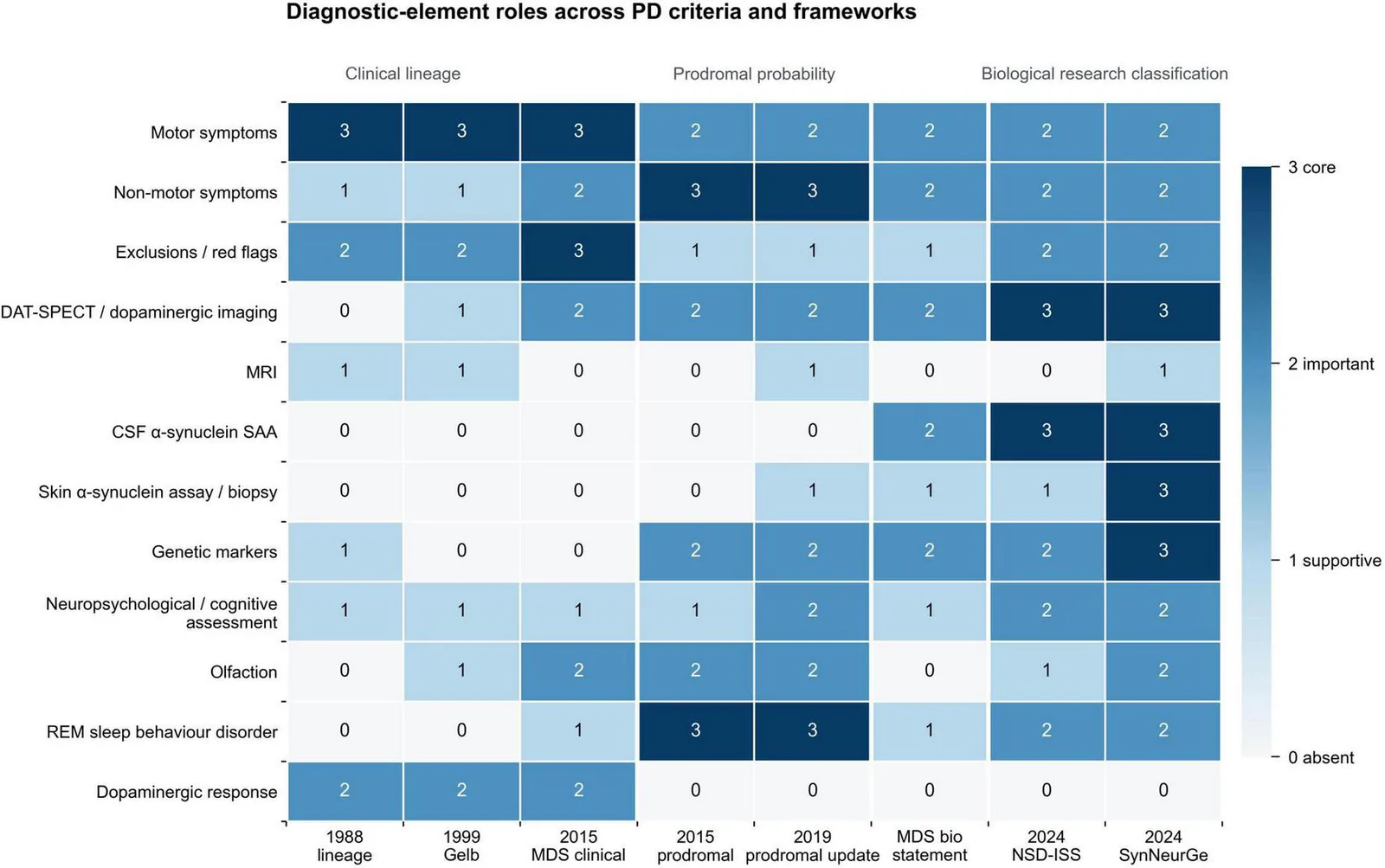
Diagnostic-element role shifts across criteria and frameworks. Heatmap of source-anchored, adjudicated diagnostic-element roles across the criteria/framework corpus. Scores indicate 0 = absent, 1 = supportive/contextual, 2 = important, and 3 = core/defining. They are interpretative ordinal judgments of textual role rather than objective measurements; category intervals are not assumed to be equal, and scores do not represent diagnostic accuracy or clinical-use endorsement. For MDS clinical criteria, dopaminergic imaging is scored for its formal exclusionary role when normal, not as a positive PD-specific biomarker. Full source notes and adjudicated scoring decisions are provided in Supplementary Table 2.

Figure 2 visualizes the same adjudicated role matrix, highlighting the shift from motor signs, exclusions, and dopaminergic response toward prodromal non-motor markers and 2024 biomarker/genetic research anchors.

### UK Brain Bank/Lewy body lineage and the clinicopathological starting point

The 1988 Gibb and Lees source was treated as a lineage/pathology anchor, not as a formal criteria-development consensus paper. It reproduces the UK Parkinson’s Disease Society Brain Bank clinical diagnostic criteria and argues that Lewy bodies provide a diagnostic marker. Its aim-like passages are therefore pathology-centered rather than process-centered. We coded them cautiously and interpreted their frequency contribution less strongly than later consensus criteria, because they are not revision aims in the same sense as Gelb et al. (1999), MDS criteria, NSD-ISS, or SynNeurGe.

The same source already anticipates later disease-boundary problems. It notes that loss of substantia nigra cells “probably starts long before symptoms develop.” It also states that “80% depletion of striatal dopamine occurs before the onset of motor symptoms.” Incidental Lewy body disease and Lewy bodies in individuals without parkinsonian features were therefore coded as delayed diagnosis (P4) and unclear presymptomatic classification (P10). In the role matrix, motor symptoms and exclusion criteria remain central to the reproduced clinical checklist, while pathology supplies the conceptual anchor. Genetics, cognition, and imaging receive only indirect or contextual scores because family history, cognitive exclusion, and CT-based structural exclusion appear without becoming positive defining criteria.

### Gelb criteria: explicit clinical diagnostic classification

Gelb et al. (1999) mark a move from clinicopathological background to explicit clinical diagnostic classification. The document notes that “few attempts have been made to develop explicit diagnostic criteria.” It then proposes “a clinical diagnostic classification” attentive to “sensitivity and specificity.” These phrases were coded as unification/simplification (A10), clinical applicability (A5), and sensitivity/specificity balancing (A1/A2). The stated problem is practical as well as epistemic: only about 75% of clinical diagnoses in cited clinicopathological series were confirmed at autopsy, and the cardinal signs of PD can occur in other conditions. P1, P3, P6, and P8 are therefore prominent.

Content scoring follows that standardization purpose. Motor signs remain core because possible and probable PD depend on Group A clinical features. Group B features and exclusions matter because they operationalize differential diagnosis. Dopaminergic response functions as a supportive probability feature rather than a defining biomarker. DAT imaging appears only at a low/supportive level in this historical context, while CSF α-synuclein SAA, skin α-synuclein assays, and modern genetic stratification are absent. The document’s main contribution is not a new disease biology. It is a more explicit way to classify clinical uncertainty.

### MDS clinical criteria 2015: calibrated clinical probability

The 2015 MDS clinical criteria systematize expert diagnostic reasoning. The abstract states that the criteria aim to “systematize the diagnostic process,” make it “reproducible across centers,” and be “applicable by clinicians” with less specialist expertise. These passages were coded as A10 and A5. The criteria also state that they were “designed to minimize” diagnostic errors, producing A1/A2/A9 assignments. The perceived-problem passages stress a familiar difficulty: full diagnostic certainty is impossible during life, and diagnostic accuracy varies when other pathologies are not recognized.

The content shift is controlled rather than revolutionary. Parkinsonism remains the entry requirement, defined around bradykinesia plus rest tremor or rigidity. The diagnostic process then relies on absolute exclusions, red flags, and supportive criteria. Non-motor manifestations become more visible than in earlier clinical criteria, but they do not displace the motor syndrome. Imaging can be supportive or exclusionary. α-synuclein SAA and skin α-synuclein assays are absent because the criteria predate their current role. External validation studies, used here for contextual interpretation rather than as primary coded evidence, show why this calibrated clinical logic mattered. In validation cohorts, the MDS criteria reached 92.6% overall accuracy for probable PD against expert clinical diagnosis and outperformed UK Brain Bank criteria in sensitivity, specificity, and overall accuracy (Postuma et al., 2015, 2018).

### MDS prodromal criteria 2015 and update 2019: probability before motor diagnosis

The 2015 MDS prodromal criteria shift the target from manifest PD to the probability of future PD. The source describes “research criteria and probability methodology for the diagnosis of prodromal PD.” It also calls the criteria a “first step in the formal delineation of early stages of PD.” These passages were coded as A3, A8, and A10. The central problem is that classic clinical diagnosis “is not yet possible” in prodromal disease, and that there is “no means to identify prodromal PD with 100% certainty.” This produced P4/P10/P3 assignments.

Role scoring changes accordingly. Non-motor and risk markers become central: RBD, olfaction, autonomic symptoms, subtle motor signs, cognition, and imaging enter the probability framework. The 2019 update reinforces this logic. It states that prodromal diagnostic criteria are “designed to be updated as new evidence becomes available.” The update also aims “to improve the accuracy of prodromal PD diagnosis.” Variable sensitivity, need for updating, limited prospective evidence, and marker applicability are identified as problems. These documents sit between clinical diagnosis and biological research classification. They are probabilistic rather than categorical disease-defining criteria; they do not diagnose routine clinical PD before symptoms, but they create a formal research language for early states and trial enrichment.

### MDS biological-definition statement: consensus principles and caution

The MDS biological-definition statement functions as a boundary-setting document. It calls for “wide international consensus in research aiming to stage or classify PD” and for “foundational principles for the development of a biological definition.” These passages yielded A7, A8, A10, and A4 assignments. The statement also presents biological staging as valuable because it could “detect disease in the earliest stages” and support therapeutic development, producing A3/A6/A8 assignments.

The perceived problems explain why this is not simply a technical upgrade. The statement notes that anatomical and biological measures of neurodegeneration have been limited *in vivo*. It also states that any framework must be useful and practical globally, and that there is “no reliable way of predicting if, when, and which clinical syndrome” will emerge. These statements produced P5, P7, P9, and P10. The document legitimizes biological-definition work while constraining its clinical interpretation. In the role matrix, SAA, dopaminergic imaging, genetics, and non-motor staging features become important. Still, the statement remains a principles document rather than an operational replacement for MDS clinical diagnosis.

### NSD-ISS 2024: biological definition and integrated research staging

NSD-ISS takes the strongest step toward a biomarker-defined research entity. It proposes to “redefine Parkinson’s disease and dementia with Lewy bodies as neuronal α-synuclein disease.” It also establishes a staging system “rooted in the biological anchors” and aims to “enable interventional trials at early disease stages.” These passages produced A4, A8, A3, and A6 assignments. The problem statements emphasize heterogeneous clinical syndromes, overlap among disorders, pathology beginning long before symptoms, and the historical inability to measure neuronal α-synuclein reliably *in vivo*.

The role matrix reflects the framework’s anchor logic. CSF α-synuclein SAA was scored as core/defining because the S anchor is based on pathological neuronal α-synuclein as detected by SAA in the current framework. DAT-SPECT/dopaminergic dysfunction was also scored as core because the D anchor is central to the research staging architecture, although this should not be read as a universally required clinical diagnostic test. Genetics was scored important rather than broadly defining, because the framework’s G anchor is narrower than a general PD-genetics category. This scoring captures a key conceptual difference: NSD-ISS tends to treat biomarker positivity as disease-defining for the research construct, while retaining explicit research-use boundaries.

### SynNeurGe 2024: multidimensional biological classification

SynNeurGe shares the biological turn but organizes it differently. It states that disease-modifying treatments could target molecular disease before symptom onset, acknowledges disease complexity and heterogeneity, and aims to enable advances in basic and clinical research. These statements yielded A3, A4, A6, and A8 assignments. Its problem statements identify exclusive reliance on clinical diagnosis without adequate biological stratification, the late appearance of motor features after substantial dopaminergic neuronal loss, and the absence of a single neurobiologically based disease construct.

The S/N/G structure explains several non-intuitive scores in Table 2. CSF α-synuclein SAA is core because the S component is central to synuclein pathology classification. Skin α-synuclein assay/biopsy was scored higher in SynNeurGe than in NSD-ISS because SynNeurGe permits a broader set of tissue-based synuclein measures within its S axis. Genetics was scored core because the G axis is a defining dimension, not a secondary risk modifier. DAT-SPECT and other neurodegeneration measures are important under the N axis. Unlike NSD-ISS, SynNeurGe reads as a multidimensional biological classification architecture in which risk, synuclein pathology, neurodegeneration, genetics, and clinical features can be combined, rather than as a single biomarker-positive disease definition.

**TABLE 2:** Diagnostic-element role scores across documents.

Diagnostic element	1988 lineage	1999 Gelb	2015 MDS clinical	2015 prodromal	2019 prodromal update	MDS bio statement	2024 NSD-ISS	2024 SynNeurGe
Motor symptoms	3	3	3	2	2	2	2	2
Non-motor symptoms	1	1	2	3	3	2	2	2
Exclusion criteria / red flags	2	2	3	1	1	1	2	2
DAT-SPECT / dopaminergic imaging	0	1	2	2	2	2	3	3
MRI	1	1	0	0	1	0	0	1
CSF α-synuclein SAA	0	0	0	0	0	2	3	3
Skin α-synuclein assay/biopsy	0	0	0	0	1	1	1	3
Genetic markers	1	0	0	2	2	2	2	3
Neuropsychological/cognitive assessment	1	1	1	1	2	1	2	2
Olfaction	0	1	2	2	2	0	1	2
REM sleep behavior disorder	0	0	1	3	3	1	2	2
Dopaminergic response	2	2	2	0	0	0	0	0

Role scale: 0 = absent; 1 = supportive/contextual; 2 = important; 3 = core/defining. Scores are interpretative document-role categories rather than quantitative measures of diagnostic value. Full source notes are provided in Supplementary Table 2.

## Discussion

### A layered diagnostic architecture

Across the selected documents, successive frameworks accumulated rather than simply displaced earlier diagnostic logics. Clinical criteria retained a patient-facing motor and differential-diagnostic function, prodromal criteria formalized probabilistic early-risk research, and biological frameworks reorganized selected research populations around molecular, imaging, and genetic anchors.

This interpretation extends prior narrative histories by adding a document-level account of what each revision was trying to solve (Marsili et al., 2018). Within the analyzed corpus, the post-2015 concentration of early-identification, biomarker, staging, and trial-readiness aims coexisted with persistent concerns about misdiagnosis and clinicopathological validity. The newer biological logic therefore appeared layered onto, rather than fully substituting for, clinical-pathological reasoning.

### Biomarkers as research anchors, not clinical substitutes

Our analysis suggests that the 2024 transition can be interpreted as a change in the functions assigned to biomarkers. SAA does not merely add a test to a clinical checklist; within NSD-ISS and SynNeurGe, it helps define or stratify the research object itself. This affects cohort construction, stage assignment, and the therapeutic trials that can be designed. In PD, however, α-synuclein, dopaminergic dysfunction, genetic risk, and clinical phenotype do not always converge into a single linear disease pathway.

The biological reclassification of Alzheimer’s disease provides a useful but incomplete comparator (Dubois et al., 2007; Jack et al., 2018). Both fields increasingly distinguish a biological research construct from the clinical syndrome and seek to identify biologically positive individuals before substantial functional impairment, partly to support earlier therapeutic trials. Alzheimer frameworks organize amyloid, tau, and neurodegeneration as partially separable biological dimensions, whereas PD frameworks must reconcile α-synuclein aggregation, dopaminergic dysfunction, heterogeneous genetic mechanisms, and clinical phenotypes that do not invariably converge. At present, α-synuclein SAA positivity identifies a biological process more readily than it predicts disease stage, progression rate, or the eventual clinical syndrome. The simultaneous emergence of NSD-ISS and SynNeurGe further distinguishes PD by leaving unresolved whether biological classification should primarily operate as an integrated staging system or as a multidimensional stratification architecture.

Recent MDS and commentary literature makes the same caution explicit. The MDS viewpoint and subsequent reviews emphasize that the biological frameworks are research constructs and are not ready for routine clinical diagnosis (Cimmino et al., 2025; Höglinger and Lang, 2024; Kalia et al., 2024; McNamara et al., 2026; Reyes et al., 2025). SAA studies show strong diagnostic promise and mechanistic relevance. They also raise questions about assay standardization, tissue source, false positives beyond classical synucleinopathies, longitudinal prediction, and access (Agin-Liebes et al., 2025; Bentivenga et al., 2024; Coughlin et al., 2025; Fernandes Gomes et al., 2023; Grossauer et al., 2023; Martinez-Valbuena et al., 2025; Orrú et al., 2025; Siderowf et al., 2023; Simuni et al., 2024; Zhao et al., 2025). Our analysis explains why these cautions are not merely technical. Once a biomarker becomes a defining criterion in research, uncertainty about that biomarker becomes uncertainty about the boundaries of the disease construct.

### Distinct architectures within the 2024 biological turn

**FIGURE 3:**
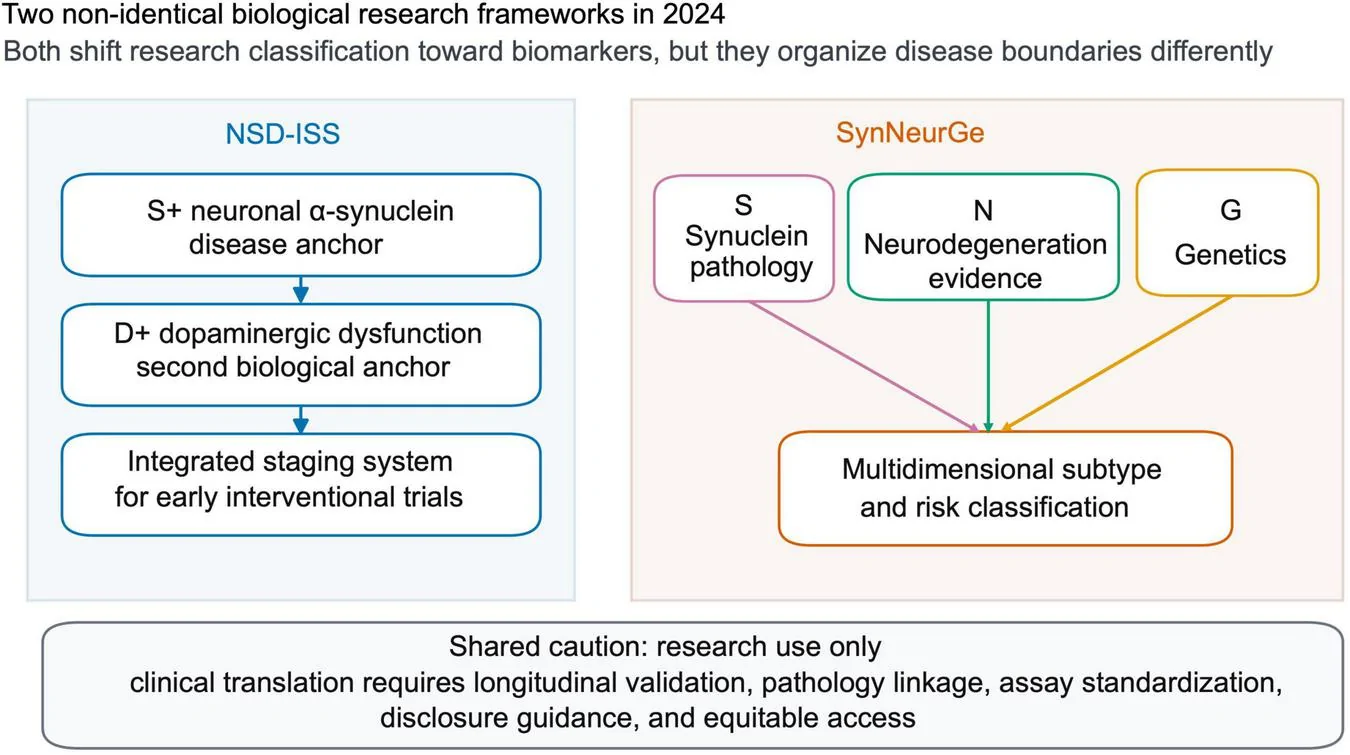
Contrasting biological research architectures in 2024. Conceptual comparison of NSD-ISS and SynNeurGe. The figure does not imply hierarchy, competition, or a clinical implementation pathway. NSD-ISS is represented as an integrated staging framework around biological anchors, whereas SynNeurGe is represented as a multidimensional S/N/G classification architecture. Both frameworks require longitudinal validation, pathology linkage, assay standardization, disclosure guidance, and equitable access before clinical translation.

The analyzed documents indicate that the two 2024 frameworks represent distinct architectures within a shared biological turn. NSD-ISS defines neuronal α-synuclein disease through biological anchors and integrated staging. In simplified terms, it treats pathological neuronal α-synuclein detection and dopaminergic dysfunction as anchors for a staged research entity. SynNeurGe uses a multidimensional S/N/G classification in which synuclein pathology, neurodegeneration, genetics, and clinical features represent heterogeneity. In our comparative reading, SynNeurGe was more explicitly plural and stratifying, whereas NSD-ISS more closely resembled an integrated disease-staging system. Figure 3 summarizes this contrast as a difference in framework architecture rather than a hierarchy or clinical implementation pathway.

This difference may have practical consequences for cohort comparability, participant communication, and how asymptomatic biomarker-positive individuals are described. It also affects genetic information: in our role matrix, genetics was important but not broadly defining in NSD-ISS (score 2), whereas it was a defining axis in SynNeurGe (score 3). These contrasting functions illustrate why future validation should specify which framework is being used for disease definition, staging, mechanistic stratification, or trial enrichment rather than assuming a single biological classification output.

### PD-specific validation and the pathology constraint

Parkinson’s disease’s diagnostic evolution is constrained by a stronger clinicopathological tradition than some biomarkerized comparators. The 1999 Gelb criteria and 2015 MDS criteria both respond to a simple problem: clinical diagnosis is imperfect during life. Modern validation studies show that this problem remains clinically meaningful. The MDS criteria achieved 92.6% overall accuracy for probable PD against expert clinical diagnosis and outperformed UK Brain Bank criteria in the validation cohort (Postuma et al., 2018). A 2025 UK Brain Bank case-control study using post-mortem diagnosis as the gold standard reported sensitivity of 99%, specificity of 86%, and accuracy of 90.96% for clinical diagnosis. Dementia with Lewy bodies was a frequent post-mortem diagnosis among clinically false-positive PD cases (di Biase et al., 2025).

These data create a double lesson. Clinical diagnosis can be highly accurate, especially in expert or later-stage contexts, so biological frameworks should not be justified by caricaturing clinical diagnosis as unreliable. Clinically meaningful error also persists, especially around overlap syndromes and pathology. Biological tools may improve classification, but only if they are validated carefully. For clinical translation, a biomarker framework cannot validate itself merely by redefining PD around the biomarker it uses. It must show how biomarker-defined categories relate to clinical trajectories, genetic subgroups, therapeutic response, and post-mortem pathology.

### Boundary expansion and participant-facing consequences

Prodromal and biological frameworks extend PD backward in time. That temporal expansion is scientifically attractive because disease-modifying therapy is most plausible before irreversible neuronal loss. It also creates stage-migration risks. The Will Rogers phenomenon describes how reclassification can alter group-level outcomes without changing any individual’s prognosis (Feinstein et al., 1985). Depending on the direction and thresholds of reclassification, outcomes may appear improved in more than one category, or the residual clinically defined cohort may appear to worsen after lower-risk individuals are reassigned to an earlier biological or prodromal group. In PD, identifying biomarker-positive or prodromal individuals before motor diagnosis could alter apparent incidence, prevalence, conversion rates, and natural-history estimates. Trial enrichment may improve, but comparisons with historical clinically diagnosed cohorts become harder.

The same boundary shift has participant-facing consequences. A research participant with positive α-synuclein SAA, RBD, or a pathogenic genetic variant may not have clinical PD, yet may experience anxiety, altered self-identity, insurance or employment concerns, and uncertainty about disclosure to family members. These concerns are amplified by global inequities in biomarker access. PD SAA and tissue-based α-synuclein assays require specialized laboratory workflows, validation, and standardization. A biologically sophisticated framework can become less globally usable if its defining tests remain unavailable outside specialized centers.

### Research infrastructure and framework development

Biological frameworks also depend on research infrastructure. PPMI and related cohorts have made it possible to study biomarkers, genetics, imaging, and progression in a coordinated way (Marek et al., 2011). Such infrastructure creates shared measures, accelerates validation, and makes new criteria thinkable. Without these cohorts, SAA and staging frameworks would remain technologically interesting but nosologically underpowered.

Infrastructure also shapes the questions the field asks. Cohorts designed around available biomarkers may privilege measurable biological axes over less accessible clinical, social, or population-diversity dimensions. Industry interest in disease-modifying therapy development can further emphasize early identification and trial enrichment. This does not invalidate biomarkerized frameworks, but it suggests that criteria evolution should be read partly as a response to research and therapeutic infrastructures, not only as a neutral reflection of disease biology.

### Toward purpose-specified stratification

The next problem for PD classification is not simply whether biological markers should enter disease definition. The harder question is how different classification outputs should be matched to different uses. Current frameworks clarify parts of this problem: prodromal criteria formalize probabilistic early identification; NSD-ISS emphasizes biological staging; SynNeurGe integrates synuclein, neurodegeneration, and genetics; and clinical criteria remain necessary for patient-facing diagnosis. These logics still leave open how propagation pattern, genetic architecture, biological stage, clinical phenotype, assignment certainty, and therapeutic mechanism should be combined when the task is prognosis or mechanism-targeted trial enrichment rather than diagnosis alone.

These distinctions may directly affect clinical-trial design. Eligibility based on an integrated biological stage may produce a different cohort from eligibility based on multidimensional S/N/G profiles, even when both are described as biologically informed. Such differences could alter baseline risk, expected progression rates, target engagement, endpoint selection, and the generalizability of treatment effects to clinically diagnosed PD. They may also determine whether SAA-negative clinical PD, incompletely penetrant genetic subgroups, or biomarker-positive individuals without functional impairment are included, excluded, or analyzed separately. Trial protocols should therefore prespecify whether a framework is being used to define the study population, enrich for progression, select a therapeutic mechanism, assign stage, or support stratified analysis.

Future systems may therefore need to be explicitly multidimensional and purpose-specified. A framework intended for clinical diagnosis should not automatically use the same thresholds or outputs as one intended for trial enrichment, prognostic stratification, or mechanism selection. Such systems would need to state whether biomarkers define disease, indicate risk, assign stage, or enrich a cohort, and whether genetic findings represent disease cause, risk modifier, or treatment target. Missing or proxy evidence should be labeled rather than silently absorbed into broad residual categories. Our analysis therefore supports a move toward stratification architectures that connect biological anchors with propagation patterns, genetic mechanisms, disease stage, and therapeutic purpose while preserving transparency about intended use.

### Limitations

This study has several limitations. The analytic corpus was targeted rather than PRISMA-screened across all retrieved PubMed records. It should therefore not be confused with a systematic review of all PD diagnostic literature or biomarker diagnostic-accuracy studies, and its code frequencies describe the selected landmark documents rather than theme prevalence across the entire field. Alternative selection strategies–including greater representation of regional guidance, technology-specific consensus statements, autonomic subdomains, or digital-biomarker literature–could yield different thematic and role-score distributions. The 1988 source is also a pathology/UK Brain Bank lineage source rather than a formal criteria-revision consensus paper; its coded passages were retained as a historical anchor but interpreted cautiously in trend summaries. Finally, the role scores are interpretative ordinal judgments of document-specific function and necessarily simplify probabilistic and multidimensional frameworks; they are not objective measurements of diagnostic value.

Generalizability is another limitation. The analysis focuses on English-language, high-impact international criteria documents and may under-represent regional diagnostic practices or validation data, including Asian populations. It discusses SAA and other biomarkers as research anchors but does not perform a technical diagnostic-accuracy review of those assays. Ethical and access implications are inferred from criteria content and existing commentary literature and require empirical study in participant, clinician, and health-system settings. Independent blinded review identified disagreements in 30 of 95 aim/problem rows and 6 of 96 role-score cells before adjudication; all role-score disagreements were adjacent one-point boundary judgments. Although the overall agreement pattern was strong, these disagreements reinforce the interpretative nature of the coding and the value of future independent replication.

## Conclusion

Within the selected corpus, our analysis did not indicate a simple replacement of clinical diagnosis by biomarkers. Instead, several purpose-specific logics operated in parallel: clinical criteria for care, prodromal criteria for early-risk research, and biological frameworks for reorganizing research cohorts around α-synuclein, neurodegeneration, and genetics. Future stratification systems will need to connect these axes to propagation patterns, stage, assignment certainty, and therapeutic purpose.

For clinical practice, the immediate recommendation is caution: biological frameworks should not be translated into routine diagnostic labeling without validated use cases, access pathways, and disclosure guidance. For researchers, the priority is independent validation of NSD-ISS and SynNeurGe against longitudinal outcomes, treatment response, and pathology. For policy makers and funders, biomarker-defined research categories require attention to global access, assay standardization, and protections for biomarker-positive individuals who do not have clinical PD. The next stage of this field is likely to be integrative rather than merely biomarker-additive. Future classification systems will need to specify when they are diagnosing, staging, prognosticating, or enriching mechanistically targeted trials.

## Data Availability

The original contributions presented in this study are included in this article/Supplementary material, further inquiries can be directed to the corresponding author.
