# Supplementary material for: Layered classification in Parkinson’s disease: a content analysis of clinical, prodromal, and biological criteria evolution
Source: Front Aging Neurosci. 2026 Aug 20;18:1935021. doi: 10.3389/fnagi.2026.1935021 (PMC13538833; doi:10.3389/fnagi.2026.1935021)
Supplement: Supplementary file 1 [file Data_Sheet_1.docx]

# Supplementary Material

Supplementary material for: Layered diagnostic classification in Parkinson’s disease: a content analysis of clinical, prodromal, and biological criteria evolution

# Supplementary Methods 1. Search and corpus report

This report documents the identification logic used for the manuscript. The study used broad PubMed verification searches to check the surrounding literature and a purposively selected corpus of landmark criteria or criteria-adjacent framework documents for primary coding. The PubMed searches were not intended to generate a PRISMA-screened systematic review of all PD diagnostic studies.

## PubMed verification searches

- Search run: 2026-07-07
- Database/interface: PubMed E-utilities esearch

| Search block | Query | Hits | Sample PMIDs |
| --- | --- | --- | --- |
| criteria_original | (“Parkinson disease”[MeSH Terms] OR “Parkinson disease”[tiab] OR “Parkinsons disease”[tiab]) AND (“diagnostic criteria”[tiab] OR diagnosis[tiab] OR “classification criteria”[tiab] OR criteria[tiab] OR guidelines[tiab] OR definition[tiab]) AND (1983:2025[pdat]) | 12046 | 41452680, 41353717, 41454540, 41370624, 41231607, 40856637, 39973493, 20301651, 41409006, 41396816 |
| commentary | (“Parkinson disease”[MeSH Terms] OR “Parkinson disease”[tiab] OR “Parkinsons disease”[tiab]) AND (“diagnostic criteria”[tiab] OR criteria[tiab]) AND (commentary[tiab] OR critique[tiab] OR limitations[tiab] OR concerns[tiab] OR letter[pt] OR editorial[pt]) AND (1983:2025[pdat]) | 197 | 41238392, 41065004, 41232780, 40762364, 41316661, 41383242, 41124650, 41203629, 41128406, 41116223 |
| biological_frameworks | (“Parkinson disease”[MeSH Terms] OR “Parkinson disease”[tiab] OR “Parkinsons disease”[tiab]) AND (biological[tiab] OR biomarker[tiab] OR “alpha-synuclein”[tiab] OR “seed amplification”[tiab] OR staging[tiab] OR classification[tiab]) AND (2020:2026[pdat]) | 8084 | 42400260, 42233523, 41657964, 42172969, 42107921, 42202499, 42128254, 41886835, 41529674, 41353717 |

## Final corpus selection

The final analytic corpus was selected to match the study question: how major criteria and criteria-adjacent frameworks have described their own aims, perceived diagnostic problems, and diagnostic-element roles. Commentary and validation papers were used to contextualize the analysis but were not coded as criteria documents.

| Included document | Document type | Inclusion rationale |
| --- | --- | --- |
| Gibb & Lees / UK Brain Bank lineage (1988) | Lineage/pathology anchor | Represents the UK Brain Bank/Lewy body clinicopathological lineage and reproduces the UKPDS Brain Bank criteria. |
| Gelb et al. (1999) | Clinical diagnostic criteria | Explicit clinical diagnostic classification using possible/probable/definite PD. |
| MDS clinical criteria (2015) | Clinical diagnostic criteria | Modern MDS clinical criteria with supportive criteria, absolute exclusions, and red flags. |
| MDS prodromal research criteria (2015) | Prodromal research criteria | Introduces formal probability methodology for prodromal PD. |
| MDS prodromal criteria update (2019) | Prodromal research criteria update | Updates prodromal marker evidence and likelihood ratios. |
| MDS biological definition statement (2023) | Biological-definition statement | Sets MDS principles for biological definition, staging, and classification. |
| NSD-ISS (2024) | Research staging/classification framework | Defines neuronal α-synuclein disease and integrated staging for research. |
| SynNeurGe (2024) | Research diagnostic/classification framework | Defines S/N/G research classification integrating synuclein, neurodegeneration, and genetics. |

## Exclusion principles

Records focused only on atypical parkinsonian syndromes, rating scales, conference abstracts, unavailable full texts, or biomarker validation without a criteria/framework role were excluded from the primary content-analysis corpus.

# Supplementary Methods 2. Diagnostic-element role scoring rules

Role scale: 0 = absent; 1 = supportive/contextual; 2 = important; 3 = core/defining.

## General rules

- Score 3 when an element is required to define, classify, stage, or positively identify the entity in that document or framework.
- For biological research frameworks, score 3 can indicate a core research-classification or staging anchor and should not be interpreted as routine clinical diagnostic endorsement.
- Score 2 when an element is a major criterion, marker, anchor, likelihood-ratio component, red-flag/exclusion category, or framework component but is not alone defining.
- Score 1 when an element is mentioned only as context, supportive evidence, indirect family history, exclusionary background, or an example rather than a positive defining component.
- Score 0 when the element is not present in the extracted criteria/framework text.
- For probabilistic prodromal criteria, high-weight markers were scored according to their role in the probability model rather than whether they were mandatory in every individual.
- For MDS clinical criteria, presynaptic dopaminergic imaging was scored for its formal exclusionary role when normal, not as a positive PD-specific biomarker.

## Source notes for role scores

| Diagnostic element | Source-anchored scoring notes |
| --- | --- |
| Motor symptoms | Gibb & Lees / UK Brain Bank lineage (1988): 3 — Table 1 Step 1 requires BRADYKINESIA and at least rigidity, rest tremor, or postural instability (lines 56-64). Ambiguity: This table is embedded in a pathology review, not newly proposed in the article.Gelb et al. (1999): 3 — Possible PD requires at least 2 of 4 Group A features; probable requires at least 3 (lines 271-299, 311-320). Ambiguity: Core motor grouping includes postural instability, unlike later MDS parkinsonism definition.MDS clinical criteria (2015): 3 — Parkinsonism is prerequisite: bradykinesia plus rest tremor or rigidity (lines 253-258; table lines 290-293). Ambiguity: Postural instability is explicitly not part of MDS parkinsonism definition (lines 284-286, 365-366).MDS prodromal research criteria (2015): 2 — Subthreshold parkinsonism and quantitative motor testing are prodromal markers (lines 314-321, 407-458). Ambiguity: Important LR-based marker, but not required in every prodromal diagnosis.MDS prodromal criteria update (2019): 2 — Subthreshold parkinsonism and quantitative motor testing remain prodromal markers (lines 160-168). Ambiguity: Motor markers are important but not mandatory.MDS biological definition statement (2023): 2 — Staging should capture the disease’s motor and non-motor aspects (lines 216-220).NSD-ISS (2024): 2 — Stage 2 includes clinical signs or symptoms that can be motor or non-motor (lines 334-340).SynNeurGe (2024): 2 — C+ motor features include bradykinesia, rigidity, or rest tremor (lines 524-531). |
| Non-motor symptoms | Gibb & Lees / UK Brain Bank lineage (1988): 1 — Dementia and autonomic failure are included in the Lewy body spectrum (lines 38-44) and severe early forms appear as exclusions (lines 77-79). Ambiguity: Positive diagnostic role is not specified.Gelb et al. (1999): 1 — Dementia, hallucinations, dysautonomia, olfaction, sensory symptoms are discussed (lines 141-180, 130-137). Ambiguity: Mostly differential/exclusionary rather than positive diagnostic markers.MDS clinical criteria (2015): 2 — Nonmotor manifestations are incorporated; absence after 5 years is a red flag (lines 111-129, 510-524). Ambiguity: Nonmotor features support/differentiate but do not replace motor parkinsonism for clinical PD.MDS prodromal research criteria (2015): 3 — Prodromal criteria can be based on nonmotor markers; RBD, olfaction, constipation, somnolence, hypotension, urinary, erectile, depression are listed (lines 132-139, 314-329, 370-420). Ambiguity: Nonmotor markers are central to prodromal diagnosis, though no single nonmotor marker is mandatory.MDS prodromal criteria update (2019): 3 — RBD, olfactory loss, constipation, somnolence, OH, urinary, erectile, depression, and cognitive deficit are listed (lines 160-177). Ambiguity: Nonmotor marker set is central but probabilistic.MDS biological definition statement (2023): 2 — H&Y is criticized because it does not capture non-motor symptoms (lines 256-259).NSD-ISS (2024): 2 — Stage 2 examples include hyposmia, RBD, cognitive impairment, constipation, dysautonomia, depression, and anxiety (lines 334-341).SynNeurGe (2024): 2 — C+ features include sensory, autonomic, sleep, and cognition categories (lines 532-543). |
| Exclusion criteria / red flags | Gibb & Lees / UK Brain Bank lineage (1988): 2 — Step 2 lists Exclusion criteria for Parkinson’s disease (lines 65-85). Ambiguity: These are UKBB criteria reproduced in the paper.Gelb et al. (1999): 2 — Group B features are suggestive of alternative diagnoses (lines 277-290). Ambiguity: Not named red flags, but functionally exclusion/differential criteria.MDS clinical criteria (2015): 3 — Diagnosis relies on absolute exclusions, red flags, and supportive criteria (lines 57-64; table lines 314-348). Ambiguity: Core to applying criteria after parkinsonism is established.MDS prodromal research criteria (2015): 1 — Risk markers alone cannot diagnose disease; alternative explanations for markers should not be applied (lines 93-98, 391-395). Ambiguity: No formal red-flag/exclusion table analogous to clinical PD criteria.MDS prodromal criteria update (2019): 1 — High-penetrance mutation carriers should not be applied to criteria; marker alternate causes require assessment (lines 99-107, 83-93). Ambiguity: No formal clinical red-flag table.MDS biological definition statement (2023): 1 — The statement stresses differential clinical diagnosis and distinguishing PD/DLB/MSA (lines 382-397).NSD-ISS (2024): 2 — The framework specifies individuals who do not have neuronal α-synuclein disease, including S− D+ categories (lines 538-580).SynNeurGe (2024): 2 — Clinical features require no other, more probable explanation, and exclusion criteria are specified for S, N, and G (lines 437-444 and 641-646). |
| DAT-SPECT / dopaminergic imaging | Gibb & Lees / UK Brain Bank lineage (1988): 0 — No DAT-SPECT/PET marker is described in the criteria text.Gelb et al. (1999): 1 — PET/SPECT striatal uptake studies are discussed as evolving diagnostic neuroimaging (lines 239-260). Ambiguity: Not included in proposed criteria; role is future/supportive only.MDS clinical criteria (2015): 2 — Normal presynaptic dopaminergic imaging is an absolute exclusion (lines 325, 472-479). Ambiguity: Abnormal DAT imaging does not qualify as supportive differentiation from atypical parkinsonism (lines 241-246).MDS prodromal research criteria (2015): 2 — Clearly abnormal dopaminergic PET/SPECT has LR+ 40 (lines 318, 472-496). Ambiguity: High-weight marker but probabilistic and not mandatory.MDS prodromal criteria update (2019): 2 — Dopaminergic PET/SPECT clearly abnormal has LR+ 43.3 (lines 119-123, 160-163). Ambiguity: High-weight marker but not mandatory.MDS biological definition statement (2023): 2 — The statement discusses dopamine transporter imaging as part of current biological proposals (lines 253-260).NSD-ISS (2024): 3 — D is a second key biological anchor, and dopamine transporter SPECT categorises D+ or D− (lines 224-227 and 273-280).SynNeurGe (2024): 3 — N+ can be endorsed by Dopaminergic PET or SPECT showing striatal dopaminergic deficit (lines 253-257). |
| MRI | Gibb & Lees / UK Brain Bank lineage (1988): 1 — Structural exclusion appears as cerebral tumour or communicating hydrocephalus on CT scan (lines 81-82). Ambiguity: CT is present; MRI is not.Gelb et al. (1999): 1 — Heavily T2-weighted MRI and putaminal signs are discussed (lines 239-248). Ambiguity: Not included in proposed criteria.MDS clinical criteria (2015): 0 — Anatomical neuroimaging is mentioned as a future possibility, not a current criterion (lines 216-219). Ambiguity: No MRI/structural marker is incorporated in the criteria.MDS prodromal research criteria (2015): 0 — MRI is not included as a prodromal criterion in the extracted text. Ambiguity: Other imaging candidates are excluded for generalizability/evidence concerns.MDS prodromal criteria update (2019): 1 — MRI approaches are promising candidates, but consensus is needed (lines 214-231). Ambiguity: Candidate role only; not added to criteria.MDS biological definition statement (2023): 0 — No operational MRI or structural-imaging role is specified in the statement text.NSD-ISS (2024): 0 — Neuromelanin-sensitive MRI is described as promising for future incorporation after validation (lines 240-247). Adjudicated final score: 0.SynNeurGe (2024): 1 — MRI markers are investigational for N+, while structural MRI can rule out atypical parkinsonism (lines 261-276). |
| CSF α-synuclein SAA | Gibb & Lees / UK Brain Bank lineage (1988): 0 — No CSF alpha-synuclein seed amplification assay appears in the extracted text.Gelb et al. (1999): 0 — No CSF alpha-synuclein SAA appears in the extracted text.MDS clinical criteria (2015): 0 — No CSF alpha-synuclein SAA appears in the extracted criteria.MDS prodromal research criteria (2015): 0 — No CSF alpha-synuclein SAA appears in the extracted criteria.MDS prodromal criteria update (2019): 0 — No CSF alpha-synuclein SAA appears in the extracted 2019 update.MDS biological definition statement (2023): 2 — SAA is discussed as a central proposed biomarker, with caution against making it the singular feature (lines 253-264).NSD-ISS (2024): 3 — Only a CSF α-synuclein seed amplification assay has undergone robust validation (lines 218-227).SynNeurGe (2024): 3 — CSF α-synuclein seed amplification assays are endorsed S+ biomarkers (lines 155-158 and 184-187). |
| Skin α-synuclein assay/biopsy | Gibb & Lees / UK Brain Bank lineage (1988): 0 — No skin alpha-synuclein assay appears in the extracted text.Gelb et al. (1999): 0 — No skin alpha-synuclein assay appears in the extracted text.MDS clinical criteria (2015): 0 — Methods to detect alpha-synuclein deposition are only future-facing (lines 216-219). Ambiguity: No skin assay criterion.MDS prodromal research criteria (2015): 0 — No skin alpha-synuclein marker appears in the extracted criteria.MDS prodromal criteria update (2019): 1 — Skin phosphorylated alpha-synuclein is promising but not yet added (lines 255-267). Ambiguity: Supportive/candidate only due lack of prospective studies.MDS biological definition statement (2023): 1 — The statement notes α-syn in other tissues such as skin but does not endorse a final criterion (lines 207-215).NSD-ISS (2024): 1 — Skin biopsy measures are promising, but NSD-ISS says only CSF SAA is currently robustly validated (lines 197-222).SynNeurGe (2024): 3 — Skin SAA and skin immunohistochemistry/immunofluorescence are endorsed S+ biomarkers (lines 155-161). |
| Genetic markers | Gibb & Lees / UK Brain Bank lineage (1988): 1 — more than one affected relative is an exclusion criterion (line 72). Ambiguity: Genetics is used only indirectly as family history/exclusion.Gelb et al. (1999): 0 — No genetic criterion is specified in the proposed criteria. Ambiguity: Familial references are not criteria content.MDS clinical criteria (2015): 0 — No genetic marker appears in the MDS clinical diagnostic criteria.MDS prodromal research criteria (2015): 2 — Family history and known genetic mutation are risk markers (lines 299-313, 339-357). Ambiguity: Genetic risk cannot diagnose prodromal PD without prodromal markers (lines 335-338).MDS prodromal criteria update (2019): 2 — GBA/LRRK2 penetrance and PRS are incorporated into calculations (lines 99-120, 183-204, 313-317). Ambiguity: High-penetrance monogenic cases are treated as distinct subgroups and excluded from applying criteria.MDS biological definition statement (2023): 2 — Pathogenic variants should be considered as part of a biological definition, classification, or staging system (lines 415-419).NSD-ISS (2024): 2 — Fully penetrant SNCA variants are sufficient for diagnosis, while other genetic variants require S+ evidence (lines 183-197 and 303-309).SynNeurGe (2024): 3 — G is a principal component, with endorsed fully penetrant and predisposition variants (lines 316-356). |
| Neuropsychological/cognitive assessment | Gibb & Lees / UK Brain Bank lineage (1988): 1 — early severe dementia with disturbances of memory, language and praxis is an exclusion (lines 77-79). Ambiguity: Cognitive testing is not a positive criterion.Gelb et al. (1999): 1 — Dementia preceding motor symptoms or early dementia is a Group B feature (lines 282-284). Ambiguity: Cognitive deficits are reviewed but not positive criteria.MDS clinical criteria (2015): 1 — FTD/PPA within 5 years is an absolute exclusion; dementia otherwise is not exclusionary (lines 318-319, 199-208). Ambiguity: Cognitive status is mainly differential/subtype context.MDS prodromal research criteria (2015): 1 — DLB/MCI handling is discussed, but prodromal DLB studies could not be included (lines 103-119). Ambiguity: Cognition is not a formal LR marker in the 2015 criteria.MDS prodromal criteria update (2019): 2 — Global cognitive deficit enters as a new marker with LR+ 1.8 (lines 27-32, 225-232). Ambiguity: Important but modest LR; not mandatory.MDS biological definition statement (2023): 1 — Mild cognitive impairment is noted as a pathologically validated clinical marker in pathology discussion (lines 354-358).NSD-ISS (2024): 2 — Cognitive impairment appears in stage 2 and cognitive functional impairment can anchor later stages (lines 334-343 and 513-517).SynNeurGe (2024): 2 — Mild cognitive impairment and dementia are C+ clinical features (lines 542-553). Adjudicated final score: 2. |
| Olfaction | Gibb & Lees / UK Brain Bank lineage (1988): 0 — No olfactory marker appears in the extracted text.Gelb et al. (1999): 1 — Olfactory abnormalities are described, but techniques are not commonly available (lines 130-137). Ambiguity: Discussed but not incorporated into the proposed criteria.MDS clinical criteria (2015): 2 — Olfactory loss can serve as a supportive ancillary criterion (lines 238-240, 313, 381-389). Ambiguity: Supportive only; not mandatory.MDS prodromal research criteria (2015): 2 — Olfactory loss is a prodromal marker with LR+ 4.0 (lines 322, 407-412). Ambiguity: Important but not required.MDS prodromal criteria update (2019): 2 — Olfactory loss LR+ increases to 6.4 (lines 119-123, 169). Ambiguity: Important marker, still probabilistic.MDS biological definition statement (2023): 0 — No extracted criterion-level role for olfaction was identified in this statement. Adjudicated final score: 0.NSD-ISS (2024): 1 — Olfactory dysfunction is listed among stage 2 non-motor signs/symptoms (lines 334-337 and 427-433).SynNeurGe (2024): 2 — Olfactory loss is listed as a sensory C+ feature (lines 532-533). Adjudicated final score: 2. |
| REM sleep behaviour disorder | Gibb & Lees / UK Brain Bank lineage (1988): 0 — No REM sleep behavior disorder marker appears in the extracted text.Gelb et al. (1999): 0 — RBD/REM sleep behavior disorder is not used in the extracted criteria.MDS clinical criteria (2015): 1 — RBD symptoms are listed among common nonmotor features whose absence is a red flag (lines 342-344, 510-524). Ambiguity: Not a positive supportive criterion in this clinical paper.MDS prodromal research criteria (2015): 3 — PSG-proven RBD has LR+ 130; questionnaire RBD is also included (lines 315-317, 374-406). Ambiguity: Very high-weight marker, but criteria remain probabilistic.MDS prodromal criteria update (2019): 3 — PSG-proven RBD remains LR+ 130; possible RBD LR+ 2.8 (lines 160-161). Ambiguity: Very high-weight marker but not mandatory.MDS biological definition statement (2023): 1 — RBD is named as a common prodromal condition among α-syn aggregation disorders (lines 372-373).NSD-ISS (2024): 2 — RBD is listed among stage 2 non-motor signs/symptoms (lines 334-341 and 427-433). Adjudicated final score: 2.SynNeurGe (2024): 2 — REM-sleep behaviour disorder is listed under sleep and Cprob+ criteria (lines 539-552). Adjudicated final score: 2. |
| Dopaminergic response | Gibb & Lees / UK Brain Bank lineage (1988): 2 — Negative levodopa response is an exclusion; excellent/long levodopa response is supportive (lines 83-96). Ambiguity: Important supportive/exclusion role but not the first-step core syndrome.Gelb et al. (1999): 2 — Possible/probable criteria require or allow sustained levodopa/dopamine agonist response; nonresponse excludes categories (lines 306-320, 335-337). Ambiguity: Important but not a first-line motor feature.MDS clinical criteria (2015): 2 — Clear dopaminergic response and dyskinesia are supportive; absence of high-dose response excludes PD (lines 306-322, 396-423, 452-467). Ambiguity: Important but not required if other supportive criteria suffice.MDS prodromal research criteria (2015): 0 — Dopaminergic treatment response is not a prodromal criterion. Ambiguity: Levodopa-responsive parkinsonism is mentioned only in DLB overlap context.MDS prodromal criteria update (2019): 0 — Dopaminergic treatment response is not used as a prodromal criterion in the extracted update.MDS biological definition statement (2023): 0 — The statement mentions treatment responsivity generally, but not dopaminergic treatment response as a PD criterion.NSD-ISS (2024): 0 — Dopamine replacement is historical background, while D status is defined by biomarkers rather than treatment response (lines 240-243 and 249-280).SynNeurGe (2024): 0 — No extracted SynNeurGe criterion uses dopaminergic treatment response; N is operationalised by imaging biomarkers (lines 253-260). |

# Supplementary Methods 3. Independent reproducibility check

## Purpose

An independent blinded reproducibility check was used to stress-test codebook clarity and identify unstable aim/problem codes or boundary role scores before final adjudication. Because this check was not performed by a second human movement-disorders specialist, it is not presented as formal human-coder reliability evidence in the main manuscript.

## Methods

For aims/problems, each quoted row was treated as a multi-label assignment and expanded into binary indicators for A1-A10 or P1-P10. Cohen’s kappa was computed separately for each binary code and summarized as aim, problem, and overall macro-average values. For diagnostic-element role scoring, each document-by-element cell was scored on the ordinal 0-3 scale and compared using unweighted, linearly weighted, and quadratically weighted kappa, alongside exact agreement and mean absolute difference. These metrics were used to identify categories requiring adjudication rather than to claim definitive coder reliability.

## Results

| Summary measure | Value |
| --- | --- |
| Aim-code macro-average kappa | 0.816 |
| Problem-code macro-average kappa | 0.838 |
| Overall A/P macro-average kappa | 0.827 |
| Exact set agreement rate, all A/P rows | 0.684 |
| Mean Jaccard similarity, all A/P rows | 0.824 |
| A/P disagreement rows | 30 / 95 |
| Role scoring quadratic weighted kappa | 0.970 |
| Role scoring linear weighted kappa | 0.944 |
| Role scoring unweighted kappa | 0.915 |
| Role scoring exact agreement rate | 0.938 |
| Role scoring mean absolute difference | 0.062 |
| Role-score disagreement cells | 6 / 96 |
| Role-score disagreements with absolute difference ≥2 | 0 |

The lowest aim-code kappas were A7, A8, and A9, indicating that cross-population applicability, staging/subtyping, and misdiagnosis-reduction aims were the least stable categories. All role-score disagreements were adjacent one-point boundary judgments.

## Interpretation

The reproducibility check supported the clarity of most coding categories while identifying several boundary judgments for conservative adjudication. It should be interpreted as a reproducibility and sensitivity exercise, not as a substitute for future independent human domain-specialist coding.

Disagreements were resolved conservatively using the quoted passage, immediate document context, or criterion-level role. Raw working disagreement logs are not part of the submission package. Final adjudicated passage-level codes, role scores, and by-document frequency distributions are reported in Supplementary Tables 1–3.

# Supplementary Table 1. Source-anchored passage-level coding (final adjudicated)

Each row is a source excerpt coded for stated aims or perceived diagnostic problems. Codes are final adjudicated codes used in the manuscript.

**Retrieval audit.** The complete screening lexicon comprised the following aim-oriented terms: aim, designed to, develop, framework, goal, intended, mission, need for, objective, proposal, propose, purpose, to enable, and to improve; and the following problem-oriented terms: caution, challenge, concern, controvers-, difficult, false positive, insufficient, lack, limitation, misdiagnos-, problem, risk of, and unclear. The Retrieval/inclusion basis column reports the exact screening term or terms present in the quoted source passage. Passages without a listed term are labelled as conceptual inclusions and were retained only when they explicitly stated an aim, intended use, limitation, or diagnostic problem. Retrieval terms were screening aids and did not determine final code assignment. Source locations refer to line numbers in the archived plain-text extractions used for coding.

| Document | Entry type | Entry ID | Quote | Source location | Retrieval/inclusion basis | Final codes | Interpretation |
| --- | --- | --- | --- | --- | --- | --- | --- |
| Gibb & Lees / UK Brain Bank lineage (1988) | Aim | 1988-A1 | Lewy bodies therefore provide a diagnostic marker | 30-31 | Conceptual inclusion (no listed trigger term) | A4 | Pathology is positioned as a specificity-enhancing diagnostic marker. |
| Gibb & Lees / UK Brain Bank lineage (1988) | Aim | 1988-A2 | as essential for the pathological diagnosis | 31-32 | Conceptual inclusion (no listed trigger term) | A4 | The paper elevates Lewy bodies to a defining pathological component of idiopathic Lewy body disease/PD. |
| Gibb & Lees / UK Brain Bank lineage (1988) | Aim | 1988-A3 | suggest a presymptomatic phase of the disease | 35-37 | Conceptual inclusion (no listed trigger term) | A3 | Incidental Lewy body disease is used to conceptualize a presymptomatic stage. |
| Gibb & Lees / UK Brain Bank lineage (1988) | Aim | 1988-A4 | inclusive pathological term of idiopathic Lewy body disease | 41-44 | Conceptual inclusion (no listed trigger term) | A8, A10 | The paper proposes a broader pathological disease-spectrum terminology. |
| Gibb & Lees / UK Brain Bank lineage (1988) | Aim | 1988-A5 | clarify the size and characteristics | 193-195 | Conceptual inclusion (no listed trigger term) | A8 | The study aims to characterize the population with incidental/presymptomatic Lewy body disease. |
| Gibb & Lees / UK Brain Bank lineage (1988) | Problem | 1988-P1 | loss of substantia nigra cells probably starts long before symptoms develop | 143-146 | Conceptual inclusion (no listed trigger term) | P4 | Clinical diagnosis occurs late relative to underlying nigral degeneration. |
| Gibb & Lees / UK Brain Bank lineage (1988) | Problem | 1988-P2 | 80% depletion of striatal dopamine occurs before the onset of motor symptoms | 144-147 | Conceptual inclusion (no listed trigger term) | P4 | Motor criteria miss a long preclinical interval. |
| Gibb & Lees / UK Brain Bank lineage (1988) | Problem | 1988-P3 | Lewy bodies in individuals without Parkinsonian features | 35-36 | Conceptual inclusion (no listed trigger term) | P10 | Asymptomatic Lewy body pathology creates an unresolved classification category. |
| Gibb & Lees / UK Brain Bank lineage (1988) | Problem | 1988-P4 | absence of Lewy bodies … excludes Parkinson’s disease | 33-35 | Conceptual inclusion (no listed trigger term) | P8 | The paper treats pathology as a gold standard that can conflict with clinical labeling. |
| Gibb & Lees / UK Brain Bank lineage (1988) | Problem | 1988-P5 | Lewy bodies occur with considerable frequency | 171-174 | Conceptual inclusion (no listed trigger term) | P10 | Incidental Lewy bodies in older populations complicate classification of presymptomatic disease. |
| Gelb et al. (1999) | Aim | 1999-A1 | propose a clinical diagnostic classification | 13-16 | Trigger term(s): propose, develop | A10, A5 | The paper aims to standardize a usable diagnostic classification. |
| Gelb et al. (1999) | Aim | 1999-A2 | regarding the sensitivity and specificity | 14-16 | Conceptual inclusion (no listed trigger term) | A1, A2 | The criteria are explicitly built around diagnostic sensitivity and specificity. |
| Gelb et al. (1999) | Aim | 1999-A3 | criteria that reliably distinguish PD from other conditions | 37-40 | Trigger term(s): goal, propose | A1, A9 | The central aim is to reduce misclassification among parkinsonian syndromes. |
| Gelb et al. (1999) | Aim | 1999-A4 | Three levels of diagnostic confidence are differentiated | 15-17 | Conceptual inclusion (no listed trigger term) | A8, A10 | Possible, probable, and definite categories formalize diagnostic certainty levels. |
| Gelb et al. (1999) | Aim | 1999-A5 | a less restrictive set of criteria | 61-64 | Conceptual inclusion (no listed trigger term) | A2 | The possible category is designed to capture likely cases with less certainty. |
| Gelb et al. (1999) | Aim | 1999-A6 | plausible, straightforward, standard criteria | 350-354 | Trigger term(s): intended, need for, propose, purpose | A5, A10 | The criteria are intended to be practical and standardized for clinical research. |
| Gelb et al. (1999) | Problem | 1999-P1 | few attempts have been made to develop explicit diagnostic criteria | 11-14 | Conceptual inclusion (no listed trigger term) | P6, P3 | Existing practice lacked explicit, reproducible criteria. |
| Gelb et al. (1999) | Problem | 1999-P2 | only about 75% of clinical diagnoses … confirmed at autopsy | 31-33 | Conceptual inclusion (no listed trigger term) | P1, P8 | Clinical diagnosis had limited specificity against pathological confirmation. |
| Gelb et al. (1999) | Problem | 1999-P3 | the cardinal signs can also occur in conditions other than PD | 33-37 | Conceptual inclusion (no listed trigger term) | P1 | The motor syndrome is not specific to idiopathic PD. |
| Gelb et al. (1999) | Problem | 1999-P4 | no individual clinical feature has sufficient sensitivity and specificity | 218-221 | Conceptual inclusion (no listed trigger term) | P1, P2 | Single signs cannot reliably define PD. |
| Gelb et al. (1999) | Problem | 1999-P5 | almost always a trade-off between sensitivity and specificity | 228-235 | Conceptual inclusion (no listed trigger term) | P1, P2 | Criteria design must balance false positives against false negatives. |
| MDS clinical criteria (2015) | Aim | 2015C-A1 | systematize the diagnostic process | 57-59 | Trigger term(s): aim | A10 | The criteria aim to make clinical diagnosis procedural and reproducible. |
| MDS clinical criteria (2015) | Aim | 2015C-A2 | reproducible across centers and applicable by clinicians | 58-60 | Trigger term(s): aim | A5, A10 | The paper targets multicenter reliability and usability by less specialized clinicians. |
| MDS clinical criteria (2015) | Aim | 2015C-A3 | designed to minimize both of these diagnostic errors | 167-169 | Trigger term(s): designed to | A1, A2, A9 | The criteria explicitly target both false-positive and false-negative diagnostic errors. |
| MDS clinical criteria (2015) | Aim | 2015C-A4 | Clinically Established PD: Maximizing specificity | 190-193 | Trigger term(s): goal | A1 | The established category prioritizes high specificity. |
| MDS clinical criteria (2015) | Aim | 2015C-A5 | Clinically Probable PD: Balancing sensitivity and specificity | 195-199 | Trigger term(s): goal | A1, A2 | The probable category balances case capture and diagnostic confidence. |
| MDS clinical criteria (2015) | Aim | 2015C-A6 | reliable ancillary test to serve as a single supportive criterion | 219-224 | Conceptual inclusion (no listed trigger term) | A4 | The criteria open a defined supportive role for validated ancillary markers. |
| MDS clinical criteria (2015) | Problem | 2015C-P1 | Full diagnostic certainty is impossible during life | 145-149 | Conceptual inclusion (no listed trigger term) | P8 | In-vivo clinical diagnosis remains imperfect against autopsy confirmation. |
| MDS clinical criteria (2015) | Problem | 2015C-P2 | Diagnostic accuracy varies considerably | 151-156 | Conceptual inclusion (no listed trigger term) | P1, P2 | Accuracy depends on disease duration, clinician expertise, age, and evolving knowledge. |
| MDS clinical criteria (2015) | Problem | 2015C-P3 | Diagnostic error can be attributable to failure to recognize other pathologies | 155-160 | Conceptual inclusion (no listed trigger term) | P1 | Misdiagnosis arises from confusing PD with other neurodegenerative or secondary parkinsonism. |
| MDS clinical criteria (2015) | Problem | 2015C-P4 | experienced clinicians can diagnose PD with greater accuracy than formal diagnostic criteria | 170-173 | Conceptual inclusion (no listed trigger term) | P3, P6 | Older formal criteria may underperform expert clinical judgment. |
| MDS clinical criteria (2015) | Problem | 2015C-P5 | an inherent conflict exists between sensitivity and specificity | 182-187 | Trigger term(s): false positive | P1, P2 | The criteria must manage the sensitivity-specificity trade-off for different uses. |
| MDS prodromal research criteria (2015) | Aim | 2015P-A1 | research criteria and probability methodology for the diagnosis of prodromal PD | 52-54 | Conceptual inclusion (no listed trigger term) | A3 | The article creates a formal method for prodromal diagnosis. |
| MDS prodromal research criteria (2015) | Aim | 2015P-A2 | first step in the formal delineation of early stages of PD | 60-63 | Trigger term(s): purpose | A3, A8 | The criteria explicitly stage early PD before clinical diagnosis. |
| MDS prodromal research criteria (2015) | Aim | 2015P-A3 | probable prodromal PD defined as 80% certainty | 61-63 | Conceptual inclusion (no listed trigger term) | A8 | Diagnostic probability thresholds standardize certainty levels. |
| MDS prodromal research criteria (2015) | Aim | 2015P-A4 | select candidates for future disease modification trials | 124-129 | Conceptual inclusion (no listed trigger term) | A6, A3 | A central use is recruitment for future neuroprotective/disease-modifying trials. |
| MDS prodromal research criteria (2015) | Aim | 2015P-A5 | motor and nonmotor clinical symptoms, clinical signs, and ancillary diagnostic tests | 56-60 | Trigger term(s): purpose | A4, A3 | The criteria integrate multiple clinical and ancillary markers into prodromal classification. |
| MDS prodromal research criteria (2015) | Aim | 2015P-A6 | can be applied for all types of markers | 632-640 | Trigger term(s): propose | A4, A10 | The Bayesian scaffold is designed to incorporate future clinical, genetic, and neuroimaging markers. |
| MDS prodromal research criteria (2015) | Problem | 2015P-P1 | classic clinical diagnosis … is not yet possible | 54-57 | Conceptual inclusion (no listed trigger term) | P4, P10 | Prodromal disease exists before standard motor diagnosis can be made. |
| MDS prodromal research criteria (2015) | Problem | 2015P-P2 | uncertainty inherent to this early-development field | 114-118 | Conceptual inclusion (no listed trigger term) | P10 | The early/prodromal domain lacks settled classification certainty. |
| MDS prodromal research criteria (2015) | Problem | 2015P-P3 | no means to identify prodromal PD with 100% certainty | 121-126 | Conceptual inclusion (no listed trigger term) | P10 | Prodromal diagnosis must remain probabilistic rather than definitive. |
| MDS prodromal research criteria (2015) | Problem | 2015P-P4 | The true prevalence of prodromal PD is unknown | 191-193 | Conceptual inclusion (no listed trigger term) | P10 | Baseline probability for prodromal PD is uncertain. |
| MDS prodromal research criteria (2015) | Problem | 2015P-P5 | limited number of prospective studies | 503-514 | Trigger term(s): limitation | P9 | Marker evidence is sparse, limiting trial-ready prodromal classification. |
| MDS prodromal criteria update (2019) | Aim | 2019-A1 | allow the diagnosis of prodromal Parkinson’s disease | 21-23 | Trigger term(s): framework | A3 | The update preserves the prodromal-diagnosis aim of the 2015 framework. |
| MDS prodromal criteria update (2019) | Aim | 2019-A2 | designed to be updated as new evidence becomes available | 22-25 | Trigger term(s): designed to, framework | A10, A4 | The framework is explicitly iterative and evidence-integrating. |
| MDS prodromal criteria update (2019) | Aim | 2019-A3 | To improve the accuracy of prodromal PD diagnosis | 63-67 | Trigger term(s): to improve, designed to, framework | A1, A2 | The update aims to improve diagnostic accuracy by adding new prospective evidence. |
| MDS prodromal criteria update (2019) | Aim | 2019-A4 | 4 new risk and prodromal markers enter the criteria | 307-310 | Conceptual inclusion (no listed trigger term) | A4, A3 | New markers are incorporated into prodromal classification. |
| MDS prodromal criteria update (2019) | Aim | 2019-A5 | practical calculator of prodromal PD probabilities | 293-297 | Trigger term(s): designed to | A5, A10 | The web calculator aims to make probabilistic research classification operational. |
| MDS prodromal criteria update (2019) | Aim | 2019-A6 | improve the diagnostic sensitivity and specificity | 319-321 | Trigger term(s): purpose | A1, A2 | The update explicitly aims to improve both sensitivity and specificity. |
| MDS prodromal criteria update (2019) | Problem | 2019-P1 | variable sensitivity of detecting prodromal PD | 62-65 | Conceptual inclusion (no listed trigger term) | P2 | Detection depends on assessment depth and timing relative to PD diagnosis. |
| MDS prodromal criteria update (2019) | Problem | 2019-P2 | diagnostic criteria need to be continually updated | 74-77 | Conceptual inclusion (no listed trigger term) | P5 | Rapidly evolving evidence makes static criteria inadequate. |
| MDS prodromal criteria update (2019) | Problem | 2019-P3 | prospective studies proving predictive value are still lacking | 262-267 | Trigger term(s): lack | P9 | Promising tissue biomarkers remain excluded because predictive evidence is insufficient. |
| MDS prodromal criteria update (2019) | Problem | 2019-P4 | marker definitions are often inconsistent | 253-256 | Conceptual inclusion (no listed trigger term) | P3, P6 | Inconsistent definitions limit integration of environmental and candidate markers. |
| MDS prodromal criteria update (2019) | Problem | 2019-P5 | LR of many single markers likely differs depending on ethnicity | 290-300 | Trigger term(s): lack | P7 | Cross-population validity and marker heterogeneity remain unresolved. |
| MDS biological definition statement (2023) | Aim | C-AIM-1 | wide international consensus in research aiming to stage or classify PD | 142-143 | Trigger term(s): aim | A10, A8 | The statement frames international consensus as necessary before biological PD staging/classification can be accepted. |
| MDS biological definition statement (2023) | Aim | C-AIM-2 | foundational principles for the development of a biological definition | 153-154 | Trigger term(s): develop, aim | A4, A8, A10 | The document aims to set principles rather than issue final criteria. |
| MDS biological definition statement (2023) | Aim | C-AIM-3 | detect disease in the earliest stages | 155-156 | Trigger term(s): framework | A3 | Staging is valued because early biological detection could create a prevention or slowing window. |
| MDS biological definition statement (2023) | Aim | C-AIM-4 | staging can also be pivotal in developing therapeutic approaches | 169-170 | Trigger term(s): develop | A6, A8 | Staging is presented as a way to stratify individuals for therapeutic development. |
| MDS biological definition statement (2023) | Aim | C-AIM-5 | globally accessible, clinically, and biologically anchored staging system | 217-218 | Conceptual inclusion (no listed trigger term) | A4, A5, A7, A8 | The statement calls for a system that joins clinical and biological anchors while remaining globally feasible. |
| MDS biological definition statement (2023) | Aim | C-AIM-6 | improve the development of disease-modifying treatments | 231-232 | Trigger term(s): develop | A6 | Biomarker-informed staging is linked to trial selection, stratification, and endpoints. |
| MDS biological definition statement (2023) | Aim | C-AIM-7 | to give specificity and to prevent over-generalization | 481 | Conceptual inclusion (no listed trigger term) | A1 | The clinical-trial discussion emphasizes specificity when applying staging systems. |
| MDS biological definition statement (2023) | Problem | C-PROB-1 | measure neurodegeneration anatomically and biologically has been limited in vivo | 163-164 | Conceptual inclusion (no listed trigger term) | P5 | Earlier PD diagnosis/staging relied on clinical observation because in-vivo biology was hard to measure. |
| MDS biological definition statement (2023) | Problem | C-PROB-2 | apply it globally in a useful and practical way | 182-183 | Conceptual inclusion (no listed trigger term) | P6, P7 | The statement treats global implementation and practicality as major barriers. |
| MDS biological definition statement (2023) | Problem | C-PROB-3 | no reliable way of predicting if, when, and which clinical syndrome | 221-222 | Conceptual inclusion (no listed trigger term) | P9, P10 | SAA positivity does not yet provide reliable prognostic classification for asymptomatic or early individuals. |
| MDS biological definition statement (2023) | Problem | C-PROB-4 | do not capture the non-motor symptoms | 258 | Conceptual inclusion (no listed trigger term) | P10, P2 | Existing H&Y staging is seen as too motor-focused and inadequate for preclinical/non-motor disease. |
| MDS biological definition statement (2023) | Problem | C-PROB-5 | no consensus on a unified biological definition based on α-syn | 289-290 | Conceptual inclusion (no listed trigger term) | P5, P10 | Biological staging is blocked by unresolved disease-definition questions. |
| MDS biological definition statement (2023) | Problem | C-PROB-6 | Currently, there is no such biomarker of change in PD | 469-470 | Conceptual inclusion (no listed trigger term) | P5, P9 | Clinical trials in pre-symptomatic stages lack validated dynamic biomarkers of biological change. |
| NSD-ISS (2024) | Aim | N-AIM-1 | redefine Parkinson’s disease and dementia with Lewy bodies as neuronal α-synuclein disease | 16-17 | Conceptual inclusion (no listed trigger term) | A10, A4 | The framework’s central move is replacing clinical-syndrome labels with a unifying biological disease construct. |
| NSD-ISS (2024) | Aim | N-AIM-2 | establishes a staging system… rooted in the biological anchors | 27-28 | Conceptual inclusion (no listed trigger term) | A4, A8 | The NSD-ISS makes S and D biomarkers the basis for staging. |
| NSD-ISS (2024) | Aim | N-AIM-3 | enable interventional trials at early disease stages | 35-37 | Trigger term(s): framework, to enable | A3, A6 | Early-stage trial enablement is stated as a core purpose. |
| NSD-ISS (2024) | Aim | N-AIM-4 | additional biomarkers as they emerge and are validated | 39 | Trigger term(s): intended | A4, A8 | The framework is designed to evolve with biomarker validation. |
| NSD-ISS (2024) | Aim | N-AIM-5 | therapeutic intervention before symptom onset | 74 | Conceptual inclusion (no listed trigger term) | A3, A6 | Biological definition is linked to prevention or halting progression before symptoms. |
| NSD-ISS (2024) | Aim | N-AIM-6 | accelerate therapeutic development in all disease stages | 99-101 | Trigger term(s): develop | A6, A8 | The staging system is justified as a drug-development framework across the disease continuum. |
| NSD-ISS (2024) | Aim | N-AIM-7 | reduce heterogeneity in clinical trials by requiring biological consistency | 671-673 | Conceptual inclusion (no listed trigger term) | A1, A6 | The authors expect biological inclusion rules to make trial cohorts more specific and homogeneous. |
| NSD-ISS (2024) | Aim | N-AIM-8 | clinically useful staging system to aid in early, accurate diagnosis | 701-702 | Trigger term(s): goal | A3, A5, A9 | Clinical utility is framed as an eventual goal, not current intended use. |
| NSD-ISS (2024) | Problem | N-PROB-1 | clinical syndromes and clinical progression are heterogenous, with overlap among disorders | 59-62 | Conceptual inclusion (no listed trigger term) | P1, P3, P10 | Clinical categories are described as overlapping and insufficiently specific for biology-based grouping. |
| NSD-ISS (2024) | Problem | N-PROB-2 | pathology begins long before any symptoms or signs | 64-65 | Conceptual inclusion (no listed trigger term) | P2, P4, P10 | The authors identify a long pre-symptomatic interval that clinical criteria cannot classify. |
| NSD-ISS (2024) | Problem | N-PROB-3 | n-αsyn could only be reliably measured post mortem | 76-77 | Conceptual inclusion (no listed trigger term) | P5, P8 | The old gold-standard dependence on post-mortem pathology delayed in-vivo biological diagnosis. |
| NSD-ISS (2024) | Problem | N-PROB-4 | MDS-UPDRS has limited sensitivity… in early disease stages | 542-543 | Conceptual inclusion (no listed trigger term) | P2, P6, P9 | Existing clinical scales may not detect early functional change needed for staging and trials. |
| NSD-ISS (2024) | Problem | N-PROB-5 | arbitrary, lacks standardised criteria for transition | 621 | Trigger term(s): lack | P3, P9, P10 | The prodromal-to-clinical boundary is presented as subjective and trial-limiting. |
| NSD-ISS (2024) | Problem | N-PROB-6 | available in a small number of qualified laboratories | 606 | Conceptual inclusion (no listed trigger term) | P5, P6, P7 | The CSF SAA anchor is not yet scalable or globally feasible. |
| SynNeurGe (2024) | Aim | S-AIM-1 | disease-modifying treatments could target the molecular basis… before the onset of symptoms | 8-10 | Trigger term(s): propose | A3, A6 | The proposal is motivated by pre-symptomatic molecular targeting for disease modification. |
| SynNeurGe (2024) | Aim | S-AIM-2 | acknowledges the complexity and heterogeneity of the disease | 10-11 | Trigger term(s): propose | A4, A8 | The three-component system is intended to classify biological heterogeneity. |
| SynNeurGe (2024) | Aim | S-AIM-3 | enable advances in both basic and clinical research | 17-18 | Trigger term(s): develop | A6, A8 | The classification is positioned as infrastructure for research and precision medicine. |
| SynNeurGe (2024) | Aim | S-AIM-4 | objective preclinical and clinical diagnosis and staging | 75-76 | Trigger term(s): objective | A3, A4, A8 | Biological classification is expected to support objective early diagnosis and subdivision. |
| SynNeurGe (2024) | Aim | S-AIM-5 | encompasses and harmonises different concepts | 118 | Conceptual inclusion (no listed trigger term) | A8, A10 | The framework unifies preclinical, prodromal, non-motor, PD, PDD, and DLB concepts under biological classification. |
| SynNeurGe (2024) | Aim | S-AIM-6 | Designed for all types of research studies | 598 | Conceptual inclusion (no listed trigger term) | A4, A6, A8 | The authors explicitly frame SynNeurGe as a broad research platform beyond one trial type. |
| SynNeurGe (2024) | Aim | S-AIM-7 | define subtypes within the broad biological spectrum | 611-612 | Conceptual inclusion (no listed trigger term) | A8 | SynNeurGe aims to subtype PD rather than impose a single sequence of disease stages. |
| SynNeurGe (2024) | Aim | S-AIM-8 | advance research on several fronts, including epidemiology… clinical trials | 749-751 | Trigger term(s): develop | A4, A6, A8 | The framework is intended to support multiple research domains, including biomarker and trial work. |
| SynNeurGe (2024) | Problem | S-PROB-1 | exclusive reliance on clinical diagnosis without adequate biological stratification | 33-34 | Conceptual inclusion (no listed trigger term) | P5, P9 | The authors link failed disease-modifying therapy development to insufficient biological stratification. |
| SynNeurGe (2024) | Problem | S-PROB-2 | motor features do not become evident until… 60–80%… neurons are lost | 67-69 | Conceptual inclusion (no listed trigger term) | P2, P4 | Clinical motor diagnosis is described as biologically late. |
| SynNeurGe (2024) | Problem | S-PROB-3 | there is no single neurobiologically based disease construct | 70-71 | Conceptual inclusion (no listed trigger term) | P5, P10 | Existing PD categories lack a unifying biological construct. |
| SynNeurGe (2024) | Problem | S-PROB-4 | Lewy pathology is neither sufficient nor necessary | 49-50 | Conceptual inclusion (no listed trigger term) | P1, P8, P10 | Clinicopathological boundaries are presented as mismatched with genetic and pathological evidence. |
| SynNeurGe (2024) | Problem | S-PROB-5 | not possible to reliably identify the disease… on the basis of clinical markers alone | 473-475 | Conceptual inclusion (no listed trigger term) | P1, P3, P10 | Early clinical markers are diverse, non-specific, and unreliable without biological evidence. |
| SynNeurGe (2024) | Problem | S-PROB-6 | limited understanding of the natural history… in asymptomatic individuals | 680-683 | Trigger term(s): concern | P9, P10 | The paper flags uncertain prognosis and ethical implications of applying criteria to asymptomatic people. |

# Supplementary Table 2. Diagnostic-element role scores and source notes (final adjudicated)

Role scale: 0 = absent; 1 = supportive/contextual; 2 = important; 3 = core/defining. Scores reflect textual role in each document, not clinical-use endorsement or diagnostic accuracy.

**Panel A. Adjudicated role matrix**

| Diagnostic element | Gibb & Lees / UK Brain Bank lineage (1988) | Gelb et al. (1999) | MDS clinical criteria (2015) | MDS prodromal research criteria (2015) | MDS prodromal criteria update (2019) | MDS biological definition statement (2023) | NSD-ISS (2024) | SynNeurGe (2024) |
| --- | --- | --- | --- | --- | --- | --- | --- | --- |
| Motor symptoms | 3 | 3 | 3 | 2 | 2 | 2 | 2 | 2 |
| Non-motor symptoms | 1 | 1 | 2 | 3 | 3 | 2 | 2 | 2 |
| Exclusion criteria / red flags | 2 | 2 | 3 | 1 | 1 | 1 | 2 | 2 |
| DAT-SPECT / dopaminergic imaging | 0 | 1 | 2 | 2 | 2 | 2 | 3 | 3 |
| MRI | 1 | 1 | 0 | 0 | 1 | 0 | 0 | 1 |
| CSF α-synuclein SAA | 0 | 0 | 0 | 0 | 0 | 2 | 3 | 3 |
| Skin α-synuclein assay/biopsy | 0 | 0 | 0 | 0 | 1 | 1 | 1 | 3 |
| Genetic markers | 1 | 0 | 0 | 2 | 2 | 2 | 2 | 3 |
| Neuropsychological/cognitive assessment | 1 | 1 | 1 | 1 | 2 | 1 | 2 | 2 |
| Olfaction | 0 | 1 | 2 | 2 | 2 | 0 | 1 | 2 |
| REM sleep behaviour disorder | 0 | 0 | 1 | 3 | 3 | 1 | 2 | 2 |
| Dopaminergic response | 2 | 2 | 2 | 0 | 0 | 0 | 0 | 0 |

**Panel B. Source-anchored scoring notes**

| Document | Element | Score | Source note |
| --- | --- | --- | --- |
| Gibb & Lees / UK Brain Bank lineage (1988) | Motor symptoms | 3 | Table 1 Step 1 requires BRADYKINESIA and at least rigidity, rest tremor, or postural instability (lines 56-64). Ambiguity: This table is embedded in a pathology review, not newly proposed in the article. |
| Gibb & Lees / UK Brain Bank lineage (1988) | Non-motor symptoms | 1 | Dementia and autonomic failure are included in the Lewy body spectrum (lines 38-44) and severe early forms appear as exclusions (lines 77-79). Ambiguity: Positive diagnostic role is not specified. |
| Gibb & Lees / UK Brain Bank lineage (1988) | Exclusion criteria / red flags | 2 | Step 2 lists Exclusion criteria for Parkinson’s disease (lines 65-85). Ambiguity: These are UKBB criteria reproduced in the paper. |
| Gibb & Lees / UK Brain Bank lineage (1988) | DAT-SPECT / dopaminergic imaging | 0 | No DAT-SPECT/PET marker is described in the criteria text. |
| Gibb & Lees / UK Brain Bank lineage (1988) | MRI | 1 | Structural exclusion appears as cerebral tumour or communicating hydrocephalus on CT scan (lines 81-82). Ambiguity: CT is present; MRI is not. |
| Gibb & Lees / UK Brain Bank lineage (1988) | CSF α-synuclein SAA | 0 | No CSF alpha-synuclein seed amplification assay appears in the extracted text. |
| Gibb & Lees / UK Brain Bank lineage (1988) | Skin α-synuclein assay/biopsy | 0 | No skin alpha-synuclein assay appears in the extracted text. |
| Gibb & Lees / UK Brain Bank lineage (1988) | Genetic markers | 1 | more than one affected relative is an exclusion criterion (line 72). Ambiguity: Genetics is used only indirectly as family history/exclusion. |
| Gibb & Lees / UK Brain Bank lineage (1988) | Neuropsychological/cognitive assessment | 1 | early severe dementia with disturbances of memory, language and praxis is an exclusion (lines 77-79). Ambiguity: Cognitive testing is not a positive criterion. |
| Gibb & Lees / UK Brain Bank lineage (1988) | Olfaction | 0 | No olfactory marker appears in the extracted text. |
| Gibb & Lees / UK Brain Bank lineage (1988) | REM sleep behaviour disorder | 0 | No REM sleep behavior disorder marker appears in the extracted text. |
| Gibb & Lees / UK Brain Bank lineage (1988) | Dopaminergic response | 2 | Negative levodopa response is an exclusion; excellent/long levodopa response is supportive (lines 83-96). Ambiguity: Important supportive/exclusion role but not the first-step core syndrome. |
| Gelb et al. (1999) | Motor symptoms | 3 | Possible PD requires at least 2 of 4 Group A features; probable requires at least 3 (lines 271-299, 311-320). Ambiguity: Core motor grouping includes postural instability, unlike later MDS parkinsonism definition. |
| Gelb et al. (1999) | Non-motor symptoms | 1 | Dementia, hallucinations, dysautonomia, olfaction, sensory symptoms are discussed (lines 141-180, 130-137). Ambiguity: Mostly differential/exclusionary rather than positive diagnostic markers. |
| Gelb et al. (1999) | Exclusion criteria / red flags | 2 | Group B features are suggestive of alternative diagnoses (lines 277-290). Ambiguity: Not named red flags, but functionally exclusion/differential criteria. |
| Gelb et al. (1999) | DAT-SPECT / dopaminergic imaging | 1 | PET/SPECT striatal uptake studies are discussed as evolving diagnostic neuroimaging (lines 239-260). Ambiguity: Not included in proposed criteria; role is future/supportive only. |
| Gelb et al. (1999) | MRI | 1 | Heavily T2-weighted MRI and putaminal signs are discussed (lines 239-248). Ambiguity: Not included in proposed criteria. |
| Gelb et al. (1999) | CSF α-synuclein SAA | 0 | No CSF alpha-synuclein SAA appears in the extracted text. |
| Gelb et al. (1999) | Skin α-synuclein assay/biopsy | 0 | No skin alpha-synuclein assay appears in the extracted text. |
| Gelb et al. (1999) | Genetic markers | 0 | No genetic criterion is specified in the proposed criteria. Ambiguity: Familial references are not criteria content. |
| Gelb et al. (1999) | Neuropsychological/cognitive assessment | 1 | Dementia preceding motor symptoms or early dementia is a Group B feature (lines 282-284). Ambiguity: Cognitive deficits are reviewed but not positive criteria. |
| Gelb et al. (1999) | Olfaction | 1 | Olfactory abnormalities are described, but techniques are not commonly available (lines 130-137). Ambiguity: Discussed but not incorporated into the proposed criteria. |
| Gelb et al. (1999) | REM sleep behaviour disorder | 0 | RBD/REM sleep behavior disorder is not used in the extracted criteria. |
| Gelb et al. (1999) | Dopaminergic response | 2 | Possible/probable criteria require or allow sustained levodopa/dopamine agonist response; nonresponse excludes categories (lines 306-320, 335-337). Ambiguity: Important but not a first-line motor feature. |
| MDS clinical criteria (2015) | Motor symptoms | 3 | Parkinsonism is prerequisite: bradykinesia plus rest tremor or rigidity (lines 253-258; table lines 290-293). Ambiguity: Postural instability is explicitly not part of MDS parkinsonism definition (lines 284-286, 365-366). |
| MDS clinical criteria (2015) | Non-motor symptoms | 2 | Nonmotor manifestations are incorporated; absence after 5 years is a red flag (lines 111-129, 510-524). Ambiguity: Nonmotor features support/differentiate but do not replace motor parkinsonism for clinical PD. |
| MDS clinical criteria (2015) | Exclusion criteria / red flags | 3 | Diagnosis relies on absolute exclusions, red flags, and supportive criteria (lines 57-64; table lines 314-348). Ambiguity: Core to applying criteria after parkinsonism is established. |
| MDS clinical criteria (2015) | DAT-SPECT / dopaminergic imaging | 2 | Normal presynaptic dopaminergic imaging is an absolute exclusion (lines 325, 472-479). Ambiguity: Abnormal DAT imaging does not qualify as supportive differentiation from atypical parkinsonism (lines 241-246). |
| MDS clinical criteria (2015) | MRI | 0 | Anatomical neuroimaging is mentioned as a future possibility, not a current criterion (lines 216-219). Ambiguity: No MRI/structural marker is incorporated in the criteria. |
| MDS clinical criteria (2015) | CSF α-synuclein SAA | 0 | No CSF alpha-synuclein SAA appears in the extracted criteria. |
| MDS clinical criteria (2015) | Skin α-synuclein assay/biopsy | 0 | Methods to detect alpha-synuclein deposition are only future-facing (lines 216-219). Ambiguity: No skin assay criterion. |
| MDS clinical criteria (2015) | Genetic markers | 0 | No genetic marker appears in the MDS clinical diagnostic criteria. |
| MDS clinical criteria (2015) | Neuropsychological/cognitive assessment | 1 | FTD/PPA within 5 years is an absolute exclusion; dementia otherwise is not exclusionary (lines 318-319, 199-208). Ambiguity: Cognitive status is mainly differential/subtype context. |
| MDS clinical criteria (2015) | Olfaction | 2 | Olfactory loss can serve as a supportive ancillary criterion (lines 238-240, 313, 381-389). Ambiguity: Supportive only; not mandatory. |
| MDS clinical criteria (2015) | REM sleep behaviour disorder | 1 | RBD symptoms are listed among common nonmotor features whose absence is a red flag (lines 342-344, 510-524). Ambiguity: Not a positive supportive criterion in this clinical paper. |
| MDS clinical criteria (2015) | Dopaminergic response | 2 | Clear dopaminergic response and dyskinesia are supportive; absence of high-dose response excludes PD (lines 306-322, 396-423, 452-467). Ambiguity: Important but not required if other supportive criteria suffice. |
| MDS prodromal research criteria (2015) | Motor symptoms | 2 | Subthreshold parkinsonism and quantitative motor testing are prodromal markers (lines 314-321, 407-458). Ambiguity: Important LR-based marker, but not required in every prodromal diagnosis. |
| MDS prodromal research criteria (2015) | Non-motor symptoms | 3 | Prodromal criteria can be based on nonmotor markers; RBD, olfaction, constipation, somnolence, hypotension, urinary, erectile, depression are listed (lines 132-139, 314-329, 370-420). Ambiguity: Nonmotor markers are central to prodromal diagnosis, though no single nonmotor marker is mandatory. |
| MDS prodromal research criteria (2015) | Exclusion criteria / red flags | 1 | Risk markers alone cannot diagnose disease; alternative explanations for markers should not be applied (lines 93-98, 391-395). Ambiguity: No formal red-flag/exclusion table analogous to clinical PD criteria. |
| MDS prodromal research criteria (2015) | DAT-SPECT / dopaminergic imaging | 2 | Clearly abnormal dopaminergic PET/SPECT has LR+ 40 (lines 318, 472-496). Ambiguity: High-weight marker but probabilistic and not mandatory. |
| MDS prodromal research criteria (2015) | MRI | 0 | MRI is not included as a prodromal criterion in the extracted text. Ambiguity: Other imaging candidates are excluded for generalizability/evidence concerns. |
| MDS prodromal research criteria (2015) | CSF α-synuclein SAA | 0 | No CSF alpha-synuclein SAA appears in the extracted criteria. |
| MDS prodromal research criteria (2015) | Skin α-synuclein assay/biopsy | 0 | No skin alpha-synuclein marker appears in the extracted criteria. |
| MDS prodromal research criteria (2015) | Genetic markers | 2 | Family history and known genetic mutation are risk markers (lines 299-313, 339-357). Ambiguity: Genetic risk cannot diagnose prodromal PD without prodromal markers (lines 335-338). |
| MDS prodromal research criteria (2015) | Neuropsychological/cognitive assessment | 1 | DLB/MCI handling is discussed, but prodromal DLB studies could not be included (lines 103-119). Ambiguity: Cognition is not a formal LR marker in the 2015 criteria. |
| MDS prodromal research criteria (2015) | Olfaction | 2 | Olfactory loss is a prodromal marker with LR+ 4.0 (lines 322, 407-412). Ambiguity: Important but not required. |
| MDS prodromal research criteria (2015) | REM sleep behaviour disorder | 3 | PSG-proven RBD has LR+ 130; questionnaire RBD is also included (lines 315-317, 374-406). Ambiguity: Very high-weight marker, but criteria remain probabilistic. |
| MDS prodromal research criteria (2015) | Dopaminergic response | 0 | Dopaminergic treatment response is not a prodromal criterion. Ambiguity: Levodopa-responsive parkinsonism is mentioned only in DLB overlap context. |
| MDS prodromal criteria update (2019) | Motor symptoms | 2 | Subthreshold parkinsonism and quantitative motor testing remain prodromal markers (lines 160-168). Ambiguity: Motor markers are important but not mandatory. |
| MDS prodromal criteria update (2019) | Non-motor symptoms | 3 | RBD, olfactory loss, constipation, somnolence, OH, urinary, erectile, depression, and cognitive deficit are listed (lines 160-177). Ambiguity: Nonmotor marker set is central but probabilistic. |
| MDS prodromal criteria update (2019) | Exclusion criteria / red flags | 1 | High-penetrance mutation carriers should not be applied to criteria; marker alternate causes require assessment (lines 99-107, 83-93). Ambiguity: No formal clinical red-flag table. |
| MDS prodromal criteria update (2019) | DAT-SPECT / dopaminergic imaging | 2 | Dopaminergic PET/SPECT clearly abnormal has LR+ 43.3 (lines 119-123, 160-163). Ambiguity: High-weight marker but not mandatory. |
| MDS prodromal criteria update (2019) | MRI | 1 | MRI approaches are promising candidates, but consensus is needed (lines 214-231). Ambiguity: Candidate role only; not added to criteria. |
| MDS prodromal criteria update (2019) | CSF α-synuclein SAA | 0 | No CSF alpha-synuclein SAA appears in the extracted 2019 update. |
| MDS prodromal criteria update (2019) | Skin α-synuclein assay/biopsy | 1 | Skin phosphorylated alpha-synuclein is promising but not yet added (lines 255-267). Ambiguity: Supportive/candidate only due lack of prospective studies. |
| MDS prodromal criteria update (2019) | Genetic markers | 2 | GBA/LRRK2 penetrance and PRS are incorporated into calculations (lines 99-120, 183-204, 313-317). Ambiguity: High-penetrance monogenic cases are treated as distinct subgroups and excluded from applying criteria. |
| MDS prodromal criteria update (2019) | Neuropsychological/cognitive assessment | 2 | Global cognitive deficit enters as a new marker with LR+ 1.8 (lines 27-32, 225-232). Ambiguity: Important but modest LR; not mandatory. |
| MDS prodromal criteria update (2019) | Olfaction | 2 | Olfactory loss LR+ increases to 6.4 (lines 119-123, 169). Ambiguity: Important marker, still probabilistic. |
| MDS prodromal criteria update (2019) | REM sleep behaviour disorder | 3 | PSG-proven RBD remains LR+ 130; possible RBD LR+ 2.8 (lines 160-161). Ambiguity: Very high-weight marker but not mandatory. |
| MDS prodromal criteria update (2019) | Dopaminergic response | 0 | Dopaminergic treatment response is not used as a prodromal criterion in the extracted update. |
| MDS biological definition statement (2023) | Motor symptoms | 2 | Staging should capture the disease’s motor and non-motor aspects (lines 216-220). |
| MDS biological definition statement (2023) | Non-motor symptoms | 2 | H&Y is criticized because it does not capture non-motor symptoms (lines 256-259). |
| MDS biological definition statement (2023) | Exclusion criteria / red flags | 1 | The statement stresses differential clinical diagnosis and distinguishing PD/DLB/MSA (lines 382-397). |
| MDS biological definition statement (2023) | DAT-SPECT / dopaminergic imaging | 2 | The statement discusses dopamine transporter imaging as part of current biological proposals (lines 253-260). |
| MDS biological definition statement (2023) | MRI | 0 | No operational MRI or structural-imaging role is specified in the statement text. |
| MDS biological definition statement (2023) | CSF α-synuclein SAA | 2 | SAA is discussed as a central proposed biomarker, with caution against making it the singular feature (lines 253-264). |
| MDS biological definition statement (2023) | Skin α-synuclein assay/biopsy | 1 | The statement notes α-syn in other tissues such as skin but does not endorse a final criterion (lines 207-215). |
| MDS biological definition statement (2023) | Genetic markers | 2 | Pathogenic variants should be considered as part of a biological definition, classification, or staging system (lines 415-419). |
| MDS biological definition statement (2023) | Neuropsychological/cognitive assessment | 1 | Mild cognitive impairment is noted as a pathologically validated clinical marker in pathology discussion (lines 354-358). |
| MDS biological definition statement (2023) | Olfaction | 0 | No extracted criterion-level role for olfaction was identified in this statement. Adjudicated final score: 0. |
| MDS biological definition statement (2023) | REM sleep behaviour disorder | 1 | RBD is named as a common prodromal condition among α-syn aggregation disorders (lines 372-373). |
| MDS biological definition statement (2023) | Dopaminergic response | 0 | The statement mentions treatment responsivity generally, but not dopaminergic treatment response as a PD criterion. |
| NSD-ISS (2024) | Motor symptoms | 2 | Stage 2 includes clinical signs or symptoms that can be motor or non-motor (lines 334-340). |
| NSD-ISS (2024) | Non-motor symptoms | 2 | Stage 2 examples include hyposmia, RBD, cognitive impairment, constipation, dysautonomia, depression, and anxiety (lines 334-341). |
| NSD-ISS (2024) | Exclusion criteria / red flags | 2 | The framework specifies individuals who do not have neuronal α-synuclein disease, including S− D+ categories (lines 538-580). |
| NSD-ISS (2024) | DAT-SPECT / dopaminergic imaging | 3 | D is a second key biological anchor, and dopamine transporter SPECT categorises D+ or D− (lines 224-227 and 273-280). |
| NSD-ISS (2024) | MRI | 0 | Neuromelanin-sensitive MRI is described as promising for future incorporation after validation (lines 240-247). Adjudicated final score: 0. |
| NSD-ISS (2024) | CSF α-synuclein SAA | 3 | Only a CSF α-synuclein seed amplification assay has undergone robust validation (lines 218-227). |
| NSD-ISS (2024) | Skin α-synuclein assay/biopsy | 1 | Skin biopsy measures are promising, but NSD-ISS says only CSF SAA is currently robustly validated (lines 197-222). |
| NSD-ISS (2024) | Genetic markers | 2 | Fully penetrant SNCA variants are sufficient for diagnosis, while other genetic variants require S+ evidence (lines 183-197 and 303-309). |
| NSD-ISS (2024) | Neuropsychological/cognitive assessment | 2 | Cognitive impairment appears in stage 2 and cognitive functional impairment can anchor later stages (lines 334-343 and 513-517). |
| NSD-ISS (2024) | Olfaction | 1 | Olfactory dysfunction is listed among stage 2 non-motor signs/symptoms (lines 334-337 and 427-433). |
| NSD-ISS (2024) | REM sleep behaviour disorder | 2 | RBD is listed among stage 2 non-motor signs/symptoms (lines 334-341 and 427-433). Adjudicated final score: 2. |
| NSD-ISS (2024) | Dopaminergic response | 0 | Dopamine replacement is historical background, while D status is defined by biomarkers rather than treatment response (lines 240-243 and 249-280). |
| SynNeurGe (2024) | Motor symptoms | 2 | C+ motor features include bradykinesia, rigidity, or rest tremor (lines 524-531). |
| SynNeurGe (2024) | Non-motor symptoms | 2 | C+ features include sensory, autonomic, sleep, and cognition categories (lines 532-543). |
| SynNeurGe (2024) | Exclusion criteria / red flags | 2 | Clinical features require no other, more probable explanation, and exclusion criteria are specified for S, N, and G (lines 437-444 and 641-646). |
| SynNeurGe (2024) | DAT-SPECT / dopaminergic imaging | 3 | N+ can be endorsed by Dopaminergic PET or SPECT showing striatal dopaminergic deficit (lines 253-257). |
| SynNeurGe (2024) | MRI | 1 | MRI markers are investigational for N+, while structural MRI can rule out atypical parkinsonism (lines 261-276). |
| SynNeurGe (2024) | CSF α-synuclein SAA | 3 | CSF α-synuclein seed amplification assays are endorsed S+ biomarkers (lines 155-158 and 184-187). |
| SynNeurGe (2024) | Skin α-synuclein assay/biopsy | 3 | Skin SAA and skin immunohistochemistry/immunofluorescence are endorsed S+ biomarkers (lines 155-161). |
| SynNeurGe (2024) | Genetic markers | 3 | G is a principal component, with endorsed fully penetrant and predisposition variants (lines 316-356). |
| SynNeurGe (2024) | Neuropsychological/cognitive assessment | 2 | Mild cognitive impairment and dementia are C+ clinical features (lines 542-553). Adjudicated final score: 2. |
| SynNeurGe (2024) | Olfaction | 2 | Olfactory loss is listed as a sensory C+ feature (lines 532-533). Adjudicated final score: 2. |
| SynNeurGe (2024) | REM sleep behaviour disorder | 2 | REM-sleep behaviour disorder is listed under sleep and Cprob+ criteria (lines 539-552). Adjudicated final score: 2. |
| SynNeurGe (2024) | Dopaminergic response | 0 | No extracted SynNeurGe criterion uses dopaminergic treatment response; N is operationalised by imaging biomarkers (lines 253-260). |

# Supplementary Table 3. Aim and problem code-frequency distributions by document (final adjudicated)

Counts are non-mutually exclusive code assignments, not unique passages.

**Panel A. Aim-code assignments**

| Code | Definition | Total assignments | By document |
| --- | --- | --- | --- |
| A1 | Improve diagnostic specificity | 9 | Gelb et al. (1999): 2; MDS clinical criteria (2015): 3; MDS prodromal criteria update (2019): 2; MDS biological definition statement (2023): 1; NSD-ISS (2024): 1 |
| A2 | Improve diagnostic sensitivity | 6 | Gelb et al. (1999): 2; MDS clinical criteria (2015): 2; MDS prodromal criteria update (2019): 2 |
| A3 | Enable early/prodromal diagnosis | 13 | Gibb & Lees / UK Brain Bank lineage (1988): 1; MDS prodromal research criteria (2015): 4; MDS prodromal criteria update (2019): 2; MDS biological definition statement (2023): 1; NSD-ISS (2024): 3; SynNeurGe (2024): 2 |
| A4 | Integrate biomarkers | 16 | Gibb & Lees / UK Brain Bank lineage (1988): 2; MDS clinical criteria (2015): 1; MDS prodromal research criteria (2015): 2; MDS prodromal criteria update (2019): 2; MDS biological definition statement (2023): 2; NSD-ISS (2024): 3; SynNeurGe (2024): 4 |
| A5 | Support clinical-practice applicability or operationalization | 6 | Gelb et al. (1999): 2; MDS clinical criteria (2015): 1; MDS prodromal criteria update (2019): 1; MDS biological definition statement (2023): 1; NSD-ISS (2024): 1 |
| A6 | Enable disease-modifying therapy development | 11 | MDS prodromal research criteria (2015): 1; MDS biological definition statement (2023): 2; NSD-ISS (2024): 4; SynNeurGe (2024): 4 |
| A7 | Improve cross-population applicability | 1 | MDS biological definition statement (2023): 1 |
| A8 | Enable staging/subtyping | 19 | Gibb & Lees / UK Brain Bank lineage (1988): 2; Gelb et al. (1999): 1; MDS prodromal research criteria (2015): 2; MDS biological definition statement (2023): 4; NSD-ISS (2024): 3; SynNeurGe (2024): 7 |
| A9 | Reduce misdiagnosis | 3 | Gelb et al. (1999): 1; MDS clinical criteria (2015): 1; NSD-ISS (2024): 1 |
| A10 | Unify/simplify criteria | 13 | Gibb & Lees / UK Brain Bank lineage (1988): 1; Gelb et al. (1999): 3; MDS clinical criteria (2015): 2; MDS prodromal research criteria (2015): 1; MDS prodromal criteria update (2019): 2; MDS biological definition statement (2023): 2; NSD-ISS (2024): 1; SynNeurGe (2024): 1 |

**Panel B. Problem-code assignments**

| Code | Definition | Total assignments | By document |
| --- | --- | --- | --- |
| P1 | Insufficient specificity/misdiagnosis | 10 | Gelb et al. (1999): 4; MDS clinical criteria (2015): 3; NSD-ISS (2024): 1; SynNeurGe (2024): 2 |
| P2 | Insufficient sensitivity/missed diagnosis | 9 | Gelb et al. (1999): 2; MDS clinical criteria (2015): 2; MDS prodromal criteria update (2019): 1; MDS biological definition statement (2023): 1; NSD-ISS (2024): 2; SynNeurGe (2024): 1 |
| P3 | Excessive subjectivity | 6 | Gelb et al. (1999): 1; MDS clinical criteria (2015): 1; MDS prodromal criteria update (2019): 1; NSD-ISS (2024): 2; SynNeurGe (2024): 1 |
| P4 | Delayed diagnosis | 5 | Gibb & Lees / UK Brain Bank lineage (1988): 2; MDS prodromal research criteria (2015): 1; NSD-ISS (2024): 1; SynNeurGe (2024): 1 |
| P5 | Biomarkers not integrated | 8 | MDS prodromal criteria update (2019): 1; MDS biological definition statement (2023): 3; NSD-ISS (2024): 2; SynNeurGe (2024): 2 |
| P6 | Criteria too complex/difficult to apply | 6 | Gelb et al. (1999): 1; MDS clinical criteria (2015): 1; MDS prodromal criteria update (2019): 1; MDS biological definition statement (2023): 1; NSD-ISS (2024): 2 |
| P7 | Insufficient cross-population validity | 3 | MDS prodromal criteria update (2019): 1; MDS biological definition statement (2023): 1; NSD-ISS (2024): 1 |
| P8 | Clinicopathological mismatch | 5 | Gibb & Lees / UK Brain Bank lineage (1988): 1; Gelb et al. (1999): 1; MDS clinical criteria (2015): 1; NSD-ISS (2024): 1; SynNeurGe (2024): 1 |
| P9 | Inadequate support for early clinical trials | 8 | MDS prodromal research criteria (2015): 1; MDS prodromal criteria update (2019): 1; MDS biological definition statement (2023): 2; NSD-ISS (2024): 2; SynNeurGe (2024): 2 |
| P10 | Unclear prodromal/asymptomatic classification | 16 | Gibb & Lees / UK Brain Bank lineage (1988): 2; MDS prodromal research criteria (2015): 4; MDS biological definition statement (2023): 3; NSD-ISS (2024): 3; SynNeurGe (2024): 4 |

## **Supplementary Figure S1**

## **Supplementary Figure S1. Text-coded aims and perceived problems in criteria evolution.** Frequency of final adjudicated aim and problem codes. Counts represent non-mutually exclusive adjudicated code assignments rather than unique passages; one passage could receive multiple codes. Supplementary Table 3 provides the corresponding by-document frequency distributions; this figure provides a visual summary.
